# Seasonal to annual ocean forecasting skill and the role of model and observational uncertainty

**DOI:** 10.1002/qj.3394

**Published:** 2018-09-28

**Authors:** Stephan Juricke, Dave MacLeod, Antje Weisheimer, Laure Zanna, Tim N. Palmer

**Affiliations:** ^1^ Mathematics and Logistics, Jacobs University, Bremen, Germany; ^2^ Department of Physics, University of Oxford, Oxford, UK; ^3^ Alfred Wegener Institute, Helmholtz Centre for Polar and Marine Research, Bremerhaven, Germany; ^4^ European Centre for Medium‐Range Weather Forecasts, Reading, UK

**Keywords:** ensembles, forecasting methods, ocean, seasonal prediction, seasonal

## Abstract

Accurate forecasts of the ocean state and the estimation of forecast uncertainties are crucial when it comes to providing skilful seasonal predictions. In this study we analyse the predictive skill and reliability of the ocean component in a seasonal forecasting system. Furthermore, we assess the effects of accounting for model and observational uncertainties. Ensemble forcasts are carried out with an updated version of the ECMWF seasonal forecasting model System 4, with a forecast length of ten months, initialized every May between 1981 and 2010. We find that, for essential quantities such as sea surface temperature and upper ocean 300 m heat content, the ocean forecasts are generally underdispersive and skilful beyond the first month mainly in the Tropics and parts of the North Atlantic. The reference reanalysis used for the forecast evaluation considerably affects diagnostics of forecast skill and reliability, throughout the entire ten‐month forecasts but mostly during the first three months. Accounting for parametrization uncertainty by implementing stochastic parametrization perturbations has a positive impact on both reliability (from month 3 onwards) as well as forecast skill (from month 8 onwards). Skill improvements extend also to atmospheric variables such as 2 m temperature, mostly in the extratropical Pacific but also over the midlatitudes of the Americas. Hence, while model uncertainty impacts the skill of seasonal forecasts, observational uncertainty impacts our assessment of that skill. Future ocean model development should therefore aim not only to reduce model errors but to simultaneously assess and estimate uncertainties.

## 1 INTRODUCTION

The ocean varies on a wide range of time‐scales. Through coupling with the atmosphere, slowly varying anomalies in ocean heat content allow some predictive skill for near‐ surface atmospheric variables, months in advance. As a consequence, seasonal forecasting systems (e.g. (Wang *et al.*, 2010; Molteni *et al.*, 2011; Arribas *et al.*, 2011; Saha *et al.*, 2014; MacLachlan *et al.*, 2015)) are able to generate skilful forecasts of monthly and seasonal average conditions. A forecast is said to be skilful if the model performs significantly better than a forecast consisting of the respective mean climatology. Prominent examples of skilful seasonal forecasts are the El Niño Southern Oscillation (ENSO; (Barnston *et al.*, 2012)) and the North Altantic Oscillation (Scaife *et al.*, 2014). The ENSO tropical Pacific sea surface temperature (SST) anomalies can be predicted with some skill for about six months in advance (Wang *et al.*, 2010; Barnston *et al.*, 2012). Since the effects of strong ENSO anomalies can be detected in many regions of the globe, skilful predictions can extend far beyond the tropical Pacific (e.g. (Palmer and Anderson, 1994)). Predictability on seasonal time‐scales also arises from other slowly varying aspects of the climate system such as the land surface and sea ice (Doblas‐Reyes *et al.*, 2013). Skilful seasonal forecasts have attracted attention from stakeholders across a variety of sectors including health, energy, and agriculture.

To achieve robust and reliable predictions of the atmosphere on seasonal to annual time‐scales, an accurate representation of the ocean becomes essential (MacLachlan *et al.*, 2015). Therefore, in addition to atmospheric variables, ocean forecasts need to be analysed and evaluated for their predictive skill (Ho *et al.*, 2013). Although the last decade has seen substantial improvements in dynamical model performance, it is still proving difficult for those dynamical models to outperform simpler and computationally less expensive statistical models (van Oldenborgh *et al.*, 2005; Barnston *et al.*, 2012; Doblas‐Reyes *et al.*, 2013). Additionally, potential predictability studies in which dynamical models try to predict themselves often claim a higher predictive skill than what is observed in actual seasonal forecasts (e.g. (Becker *et al.*, 2014)). In these studies models tend to be overconfident in their ability to produce skilful predictions; a result of model‐specific systematic errors and an insufficient representation of model uncertainty. For the sake of completeness, however, it should be noted that counterexamples to such studies exist as well, where forecasts tend to have higher skill than expected from potential predictability studies (Eade *et al.*, 2014; Kumar *et al.*, 2014).

Seasonal forecasting is an inherently probabilistic problem (Palmer and Anderson, 1994). It cannot be solved by a single integration, from a single initial condition with one fixed model configuration. Uncertainties arising from model error grow considerably on the monthly time‐scale and need to be accounted for. Due to the length of the forecast, this becomes even more important for seasonal than short‐term weather forecasting. The emphasis is no longer on accurately estimating atmospheric initial condition uncertainty. Uncertainties in the ocean, land surface, and sea ice initial states of fully coupled seasonal forecasting models need to be accounted for, as well as uncertainties in the model set‐up and construction, for all relevant components. In weather forecasting, incorporating model uncertainty estimates has already led to significant improvements of forecasts (e.g. the Stochastically Peturbed Parametrized Tendency, SPPT, scheme described in (Buizza *et al.*, 1999), with the most recent version discussed in Leutbecher *et al.*, 2017. Stochastic schemes are able to substantially improve reliability, i.e. the balanced ratio between forecast error and ensemble forecast spread, by increasing underdispersive ensemble spread and simultaneously decreasing forecast error (Palmer *et al.*, 2005). The implementation of stochastic schemes also led to an increase in skill scores. In accordance with weather prediction, similar improvements hold for seasonal forecasts (Weisheimer *et al.*, 2014; Batté and Doblas‐Reyes, 2015). In this context the incorporation of stochastic schemes as a way to represent unresolved sub‐grid variability has also led to decreased model biases (Weisheimer *et al.*, 2014). In a recent study, Andrejczuk *et al.*s*(2016) extended the atmospheric SPPT approach to the ocean model component, showing improvements in reliability of upper 300 m heat content, especially at the end of 3‐month forecasts.

Stochastic schemes targeting uncertainties in other components of the climate system show promise, though they are not yet used operationally. For example Brankart (2013); Brankart *et al.*s*(2015); Cooper and Zanna (2015); Grooms (2016); Williams *et al.*s*(2016); Juricke *et al.* (2017); Cooper (2017) and Zanna *et al.*s*(2017) have investigated the impact of stochastic schemes in the ocean, MacLeod *et al.*s*(2016) in the land surface, Williams (2012) in ocean–atmosphere surface coupling, Juricke *et al.*s*(2013), Juricke and Jung (2014), and Juricke *et al.*s*(2015) in sea ice, and Ollinaho *et al.*s*(2017) in the atmosphere. Also Berner *et al.*s*(2017) gives an overview and outlook of stochastic parametrization approaches. The European Centre for Medium‐Range Weather Forecasts (ECMWF) recently described their future plans for dealing with forecast uncertainty, from the medium range to seasonal forecasts (Leutbecher *et al.*, 2017), highlighting the importance of improving the representation of model uncertainties in all model components.

Enhanced model resolution and increased model complexity can lead to improved simulations and better predictions but must be constrained and developed through the extensive use of observational data (Alves *et al.*, 2004; Vidard *et al.*, 2007; Balmaseda *et al.*, 2009; Balmaseda and Anderson, 2009). Assimilating ocean observations is essential for the generation of adequate initial conditions and estimates of initial condition uncertainty, and a topic of ongoing research. We refer the reader to Mogensen *et al.*s*(2012) and Waters *et al.*s*(2015) for data assimilation developments regarding the Nucleus for European Modelling of the Ocean (NEMO) model used in this study, or e.g. Martin *et al.*s*(2015) for a more general comparison of ocean data assimilation approaches. Models rely in their verification and evaluation on reanalysis products which assimiliate the spatially and temporally sparse observational data into the model to constrain the model dynamics. Unfortunately, large regions of both the surface and subsurface ocean remain insufficiently constrained, due to poor spatial and temporal resolution of the observations. The resulting reanalyses are therefore strongly model‐ and assimilation method‐dependent. This has been shown in a reanalysis intercomparison (Balmaseda *et al.*, 2015), for example for the meridional overturning streamfunction in the North Atlantic (Karspeck *et al.*, 2017) where reanalysis products widely differ in their estimate of the mean state and interannual variability. Observational and reanalysis uncertainty is therefore an issue that cannot be ignored, as it limits the precision of model performance evaluation and objective judgement of model development.

In this study we will analyse seasonal to annual coupled ten‐month forecasts with an emphasis on the ocean model performance. We will investigate the importance of observational uncertainty in the verification process by comparing the forecasts with two different reanalysis products. Furthermore, we will investigate how incorporation of model uncertainty estimates in the ocean model impacts forecast skill.

The paper is structured as follows: section 2 describes the forecasting system, the experimental set‐up, and the diagnostics used for verification. In section 3 the general performance of the forecasts in terms of reliability and skill is discussed. The impact of observational uncertainty on forecast skill and reliability is investigated in section 4. Section 5 deals with the improvements observed when parametrization uncertainty estimates are incorporated in the ocean model. Finally, section 6 summarizes the results.

## 2 SEASONAL FORECASTING SYSTEM

### 2.1 Model set‐up

The forecasting system used for this study is an intermediate model cycle between the ECMWF seasonal forecasting model System 4 (version c36r4; (Molteni *et al.*, 2011)) and the new seasonal forecasting system SEAS5 (version c43r1). It consists of the Integrated Forecasting System (IFS, version c41r1; (Weisheimer *et al.*, 2017)) modelling the atmospheric circulation, which includes the land surface model HTESSEL. IFS is coupled to the ocean model NEMO (Madec, 2008). The resolution of the IFS is T255 (≈80 km) in the horizontal with 91 vertical levels. NEMO has a 1° horizontal resolution with refinement to 1/3° in the Tropics and 42 vertical layers (i.e. non‐eddy resolving outside the tropical belt). Molteni *et al.*s*(2011) give further details of the model set‐up.

### 2.2 Forecast set‐up and diagnostics

The assessed hindcast period in this study ranges from 1981 to 2010, with start dates 01 May and a forecast length of ten months, covering May until February. Each start date has 20 ensemble members which are initialized with different ocean states based on the five‐member reanalysis ensemble of the ECMWF 1° ORAS4 ocean renanalysis product (Balmaseda *et al.*, 2013), with additional stochastic temperature perturbations in the upper ocean layers (Molteni *et al.*s*(2011) give details). Atmospheric initial conditions are from ERA‐Interim (Dee *et al.*, 2011) and singular vectors are used for initial condition perturbations. The atmospheric model uses two stochastic schemes: SPPT (mentioned in the previous section; (Buizza *et al.*, 1999)) and a stochastic backscatter implementation (Shutts, 2005; Berner *et al.*, 2009).

Two different experiments were carried out with this set‐up. One ensemble has no stochastic perturbations in the ocean model (henceforth called REF) except for the initial condition perturbations, while the other (called STO) applies stochastic perturbations to three ocean parametrizations.

The three schemes perturbed are:

1. the Gent–McWilliams parametrization for eddy induced advection;
2. the enhanced vertical diffusion used in cases of unstable stratification;
3. the turbulent kinetic energy (TKE) scheme used to define vertical diffusivity and viscosity;

The exact set‐up and motivation for these schemes has been described by Juricke *et al.*s*(2017). The pertubations are designed to estimate uncertainties related to these schemes, either to some specific parameter (which is the case for 1 and 2) or related to specific tendencies within the parametrization (which is the case for 3). As discussed by Juricke *et al.*s*(2017), each of these parametrization perturbations acts in very specific regions of the ocean. While 1 has a strong impact in the Southern Ocean and the western boundary currents, 2 is especially active at the deep convection sites in the high latitudes, and 3 acts in the upper ocean in the Tropics and also the western boundary currents.

To diagnose the quality of the forecasts, different diagnostics have been applied. These include the computation of atmospheric and oceanic biases as well as debiased diagnostics of the ensemble spread and ensemble mean error, and also some probabilistic skill scores. The main diagnostics presented in this paper are listed in Table 1. All diagnostics are based on monthly or seasonal means. Except for the bias, all diagnostics also use anomalies with respect to the reanalysis or model climatology.

**Table 1:** Main diagnostics presented in this paper. Upper‐case variables X and O mark total fields for model and reanalysis, respectively. Lower‐case variables x and o mark anomalies with respect to the respective climatology. Diagnostics are carried out for a specific 1‐ or 3‐month average. Overbars represent ensemble means. The number of ensemble members is M, number of start years is N, any specific model variable under consideration is denoted by x, and o are the respective observations/observational estimates. For the probabilistic Brier skill score, p defines the probability of the ensembles members (p ^x^) or the observations (p ^o^) to be below their respective climatological median. The angle brackets denote averages with respect to time, i.e. <o> is the time mean of the observations (climatology) and <p ^o^> is the time mean probability to be below the median for the observations (which is exactly 0.5).

**Name**	**Formula**
Model anomaly (year *n*)	$x_{n}=X_{n}-\frac{1}{N}\sum_{i=1}^{N}\left(\frac{1}{M}\sum_{j=1}^{M}X_{ij}\right)=X_{n}-\frac{1}{N}\sum_{i=1}^{N}\bar{X_{i}}$
Observational anomaly (year *n*)	$o_{n}=O_{n}-\frac{1}{N}\sum_{i=1}^{N}O_{i}$
BIAS	$\frac{1}{N}\sum_{i=1}^{N}\left(\frac{1}{M}\sum_{j=1}^{M}(X_{ij}-O_{i})\right)=\frac{1}{N}\sum_{i=1}^{N}(\bar{X_{i}}-O_{i})$
Mean Ensemble SPREAD	$\frac{1}{N}\sum_{i=1}^{N}\left(\sqrt{\frac{1}{M-1}\sum_{j=1}^{M}{\left(x_{ij}-\bar{x_{i}}\right)}^{2}}\right)$
Mean RMSE (Root Mean Square Error)	$\frac{1}{N}\sum_{i=1}^{N}\left(\sqrt{\frac{1}{M}\sum_{j=1}^{M}{\left(x_{ij}-o_{i}\right)}^{2}}\right)$
MSSS (Mean Squared Skill Score)	$1-\text{RMSE}/\left(\frac{1}{N}\sum_{i=1}^{N}{\left(o_{i}-<o>\right)}^{2}\right)$
BSS (Brier Skill Score)	$1-\left(\frac{1}{N}\sum_{i=1}^{N}{\left(p_{i}^{x}-p_{i}^{o}\right)}^{2}\right)/\left(\frac{1}{N}\sum_{i=1}^{N}{\left(p_{i}^{o}-<p^{o}>\right)}^{2}\right)$
	$=1-4\left(\frac{1}{N}\sum_{i=1}^{N}{\left(p_{i}^{x}-p_{i}^{o}\right)}^{2}\right)$

Although the biases for all variables discussed here have been computed and analysed, we will mostly focus on the debiased ensemble information from the seasonal forecasts. Especially for the discussion of the effects of observational and model uncertainty on the seasonal forecasts (sections 4 and 5), the effects on model biases for most variables have been more or less inconclusive.

One of the first aspects that needs to be analysed in the context of ensemble weather and seasonal forecasts is the forecast reliability, i.e. the ratio between root mean square error (RMSE) and mean ensemble standard deviation (SPREAD). A reliable ensemble forecast captures the RMSE with the SPREAD. It therefore adequately accounts for forecast uncertainty related to the chaotic, unpredictable behaviour of the climate system as well as the additional sources of uncertainty such as initial condition or model uncertainty. While a single forecast is very unlikely to remain close to the true system with increasing lead time, an ensemble system should ideally account for all possible deviations from the true state and hence balance RMSE with SPREAD.

A probabilistic diagnostic that makes use of the information provided by all the ensemble members is the Brier skill score (BSS). In the case of this study, the BSS looks at how well the forecasted likelihood of an anomaly to be above (or below) the climatological median compares to the actual occurance of such an anomaly. The median here is always based on the respective climatology, i.e. model or reference reanalysis (cf. Table 1).

Bootstrapping of the data (sample size 1,000, with replacement) is used for significance testing of the BSS and mean squared skill score (MSSS), RMSE, and SPREAD, and for the significance testing of differences in those diagnostics between the stochastic and deterministic ensembles. As reference data (i.e. truth) for the ocean diagnostics the two ECMWF ocean reanalysis products ORAS4 (Balmaseda *et al.*, 2013) and ORAP5 (Zuo *et al.*, 2017) are used. For ORAS4, which is a five‐member reanalysis and based on the ECMWF seasonal forecasting model System 4, only the unperturbed member is used as reference truth. ORAP5 is the preliminary version of the new ocean reanalysis based on the ECMWF seasonal forecasting model SEAS5 and consists of only one member. The two reanalysis products differ especially in the horizontal and vertical resolution of the ocean model, with 1° compared to 1/4° horizontal resolution and 42 vertical levels compared to 75 for ORAS4 and ORAP5, respectively. In accordance with the seasonal forecasts, both reanalyses were generated with NEMO. Therefore, it is a relatively optimistic but also fair comparison between the forecast and the reanalyses when all are based on a similar model structure. However, as discussed in Juricke *et al.*s*(2017) for low‐frequency variability of sea surface height and overturning streamfunctions, and by Karspeck *et al.*s*(2017) for the overturning streamfunction mean and variability in the North Atlantic, different reanalysis products often differ considerably. This is especially true for any local, depth‐integrated or subsurface variables that are less well constrained by the assimilated observational estimates.

For the atmospheric diagnostics, ERA‐Interim (Dee *et al.*, 2011) was used as reference data.

## 3 SEASONAL TO ANNUAL OCEAN FORECASTS

### 3.1 Forecast spread, error, and reliability

**Figure 1:**
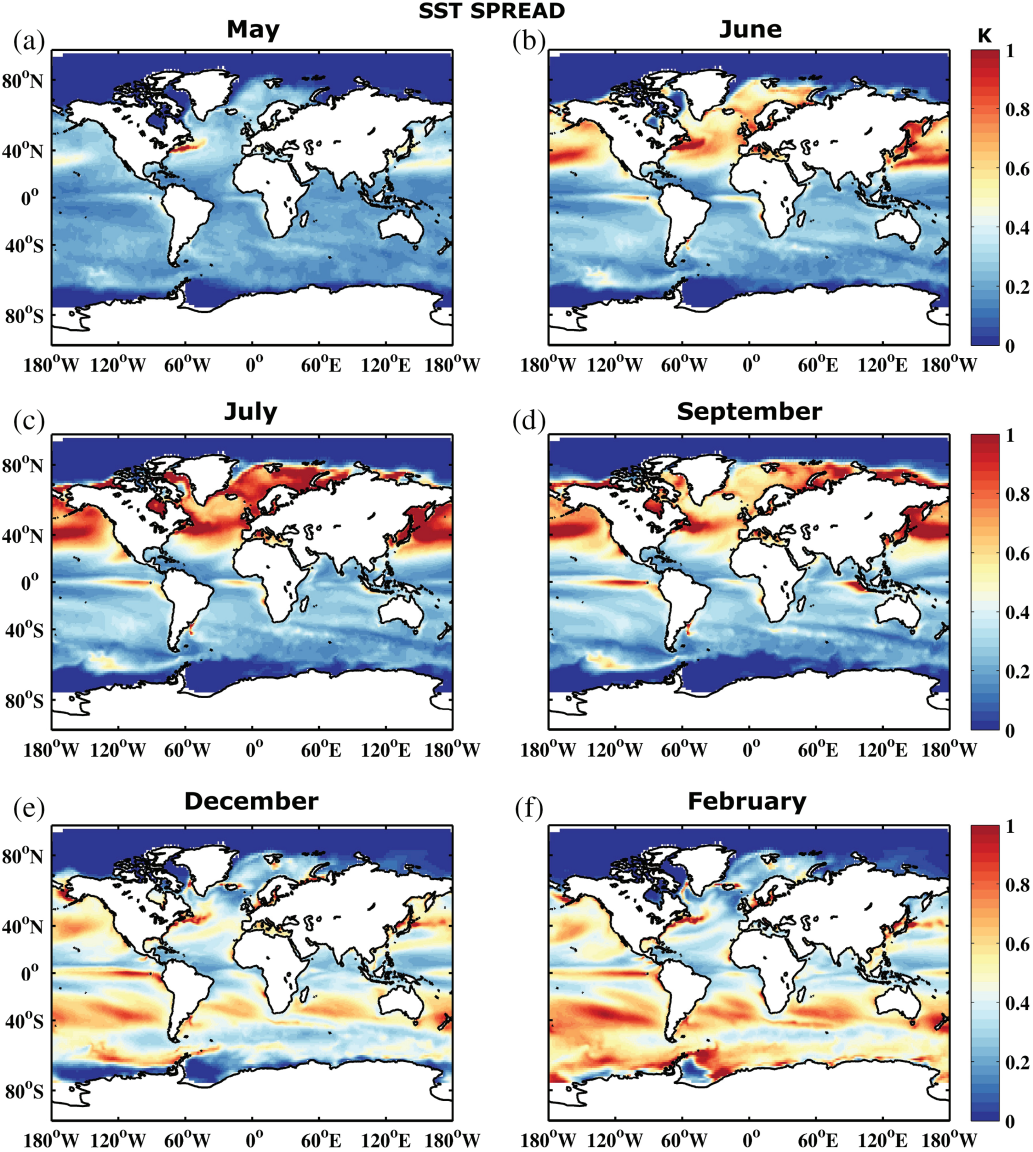
Sea surface temperature ensemble spread (K) of the reference forecast REF averaged over the 30 start dates 1981–2010 for months (a) May, (b) June, (c) July, (d) September, (e) December and (f) February

The change of SPREAD for the reference forecast with increasing lead time is illustrated in Figure 1 for SST. SPREAD increases most rapidly in turbulent regions such as the western boundary currents and, to some degree, the Southern Ocean, and in regions of strong coupling such as the Tropics. It also has a very strong seasonal signal, with a more rapid SPREAD increase in the Northern Hemisphere during the first three months. Here SPREAD seems to saturate in July (Figure 1c), especially in the midlatitudes. In general the SPREAD development in the midlatitudes follows the seasonal cycle of mixed‐layer depth. Shoaling of mixed‐layer depth in the summer hemisphere increases the impact of the atmosphere on SST variability, therefore enhancing SST SPREAD. On the other hand, deepening of the mixed layer in the winter hemisphere reduces the atmospheric influence and decreases SST SPREAD. The Tropics show a more consistent, continuous increase in spread, i.e. the impact of the seasonal cycle is strongly reduced. The Subtropics develop less ensemble spread, while the very high latitudes are strongly constrained by the disappearence/reappearence of sea ice. It should be noted that we capture spread evolution on the monthly time‐scale. On interannual time‐scales, more subtle increases in spread over the years will be related to slow modes of variability. As a consequence, the apparent saturation followed by decline in spread in the Northern Hemisphere does not signify the true climatological spread of the system, as interannual variations are not yet captured. Previous studies have shown that there is potential skill in predicting low‐frequency anomalies in, for example, the North Atlantic (e.g. (Zanna, 2012; Huddart *et al.*, 2016)).

**Figure 2:**
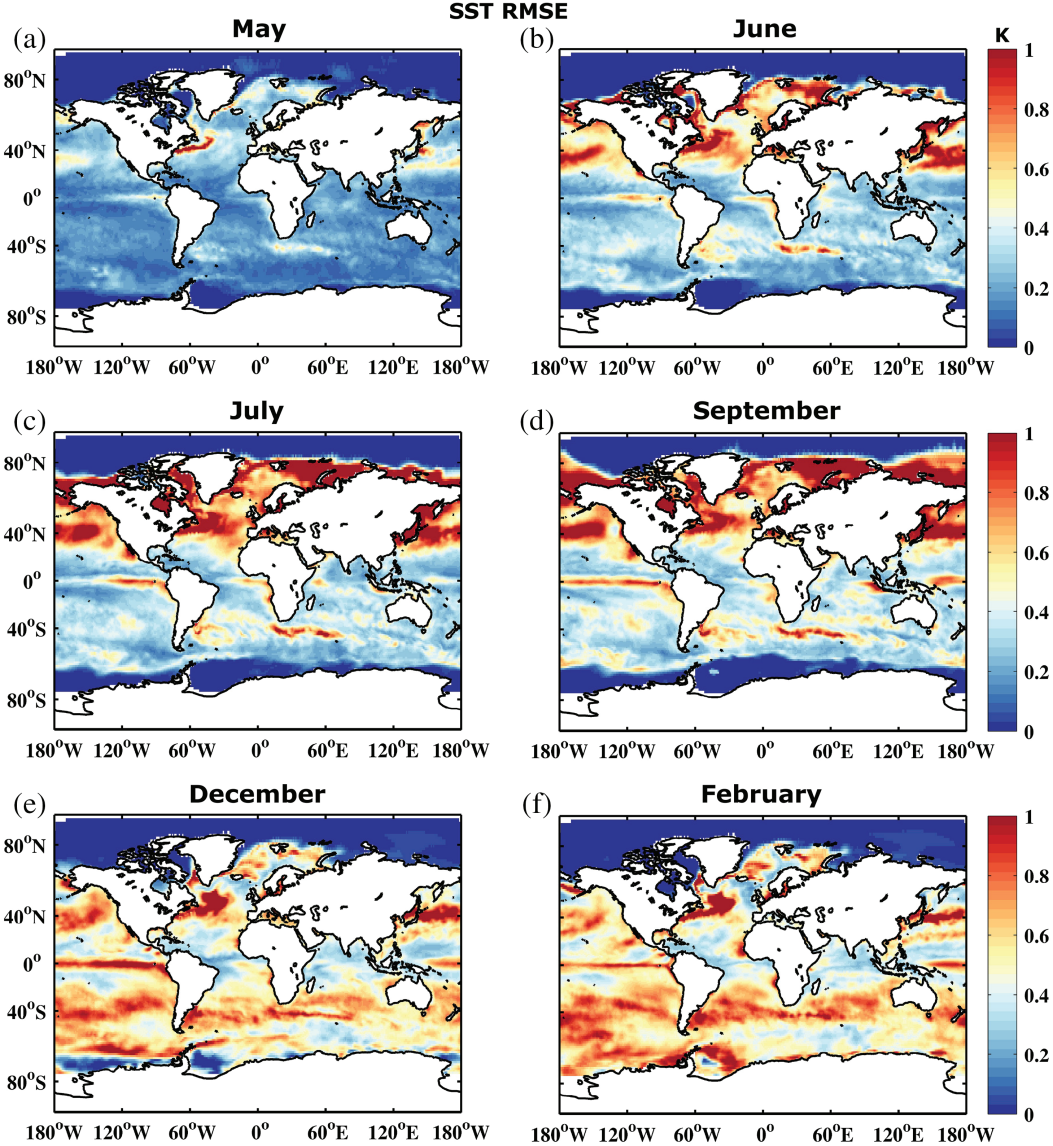
Root mean square error of sea surface temperature (K) between the ensemble mean of the reference forecast REF and the ORAS4 1° reanalysis for years 1981–2010 and months (a) May, (b) June, (c) July, (d) September, (e) December and (f) February

**Figure 3:**
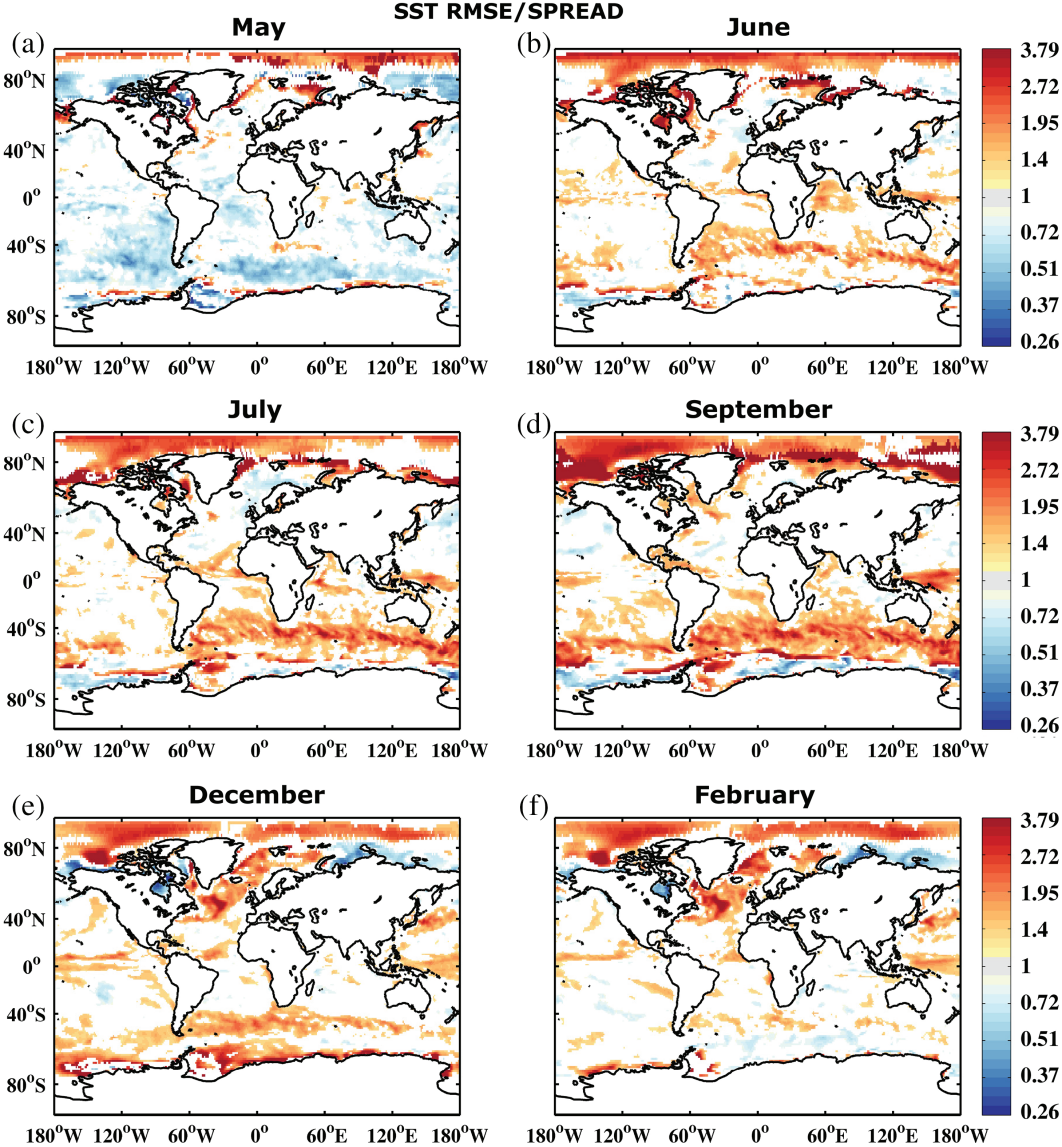
Ratio between root mean square error (referenced to ORAS4 1° reanalysis) and mean ensemble spread of sea surface temperature of the reference forecast REF for start dates 1981–2010 and months (a) May, (b) June, (c) July, (d) September, (e) December and (f) February. Areas shown are only those which are significantly different from 1 at 95% confidence according to a 1,000 sample bootstrapping with replacement. Note the nonlinear colour scale

Figure 2 shows the development of the RMSE. The error shows a similar behaviour to the SPREAD, growing at a similar rate and reflecting a more or less reliable system. However, when comparing the actual RMSE/SPREAD ratio in Figure 3, it becomes apparent that the SPREAD is smaller than the RMSE, i.e. the system is underdispersive. This is especially true in those areas where the error growth is largest, suggesting that the forecasting system is not active enough in turbulent ocean regions. To some degree this is related to the coarse ocean model resolution that does not resolve mesoscale ocean eddies (e.g. (Juricke *et al.*, 2017)). The first month, however, shows too large a SPREAD, i.e. large regions are overdispersive. A possible explanation for this is that the initial condition spread of the system is initially too large when compared to the ORAS4 reanalysis. While the seasonal forecast is initialized based on the five‐member reanalysis ensemble, the verification of the forecast for the RMSE is carried out with the unperturbed ORAS4 member. The overdispersion is therefore more or less an artifact of the choice of the reference data. We will discuss this again in section 4.1.

Similar to the RMSE and SPREAD, the ratio between the two exhibits some seasonal dependence. However, the amplitude of the seasonality is reduced and shows a clear phase shift. RMSE is significantly larger than SPREAD in the Northern Hemisphere in February (month 10, boreal winter), compared to June and July (months 2 to 3, boreal summer), while September (month 5, austral winter) shows the largest underdispersion in the Southern Hemisphere. As discussed for SPREAD, this is again related to the reduced impact of atmospheric variability and increased impact of ocean dynamics in the winter hemisphere, suggesting an insufficiently calibrated ocean model component at non‐eddy‐resolving 1° resolution.

The analysis in this study is based on forecasts initialized in May. Due to the strong seasonal SPREAD and RMSE dependence, forecasts initialized in a different month may produce different results. However, strong seasonal SPREAD dependence and the large growth in SPREAD in the first 3 months in the summer hemisphere for SST was also observed by Andrejczuk *et al.*s*(2016) with 01 November initialization (see their supplementary material).

**Figure 4:**
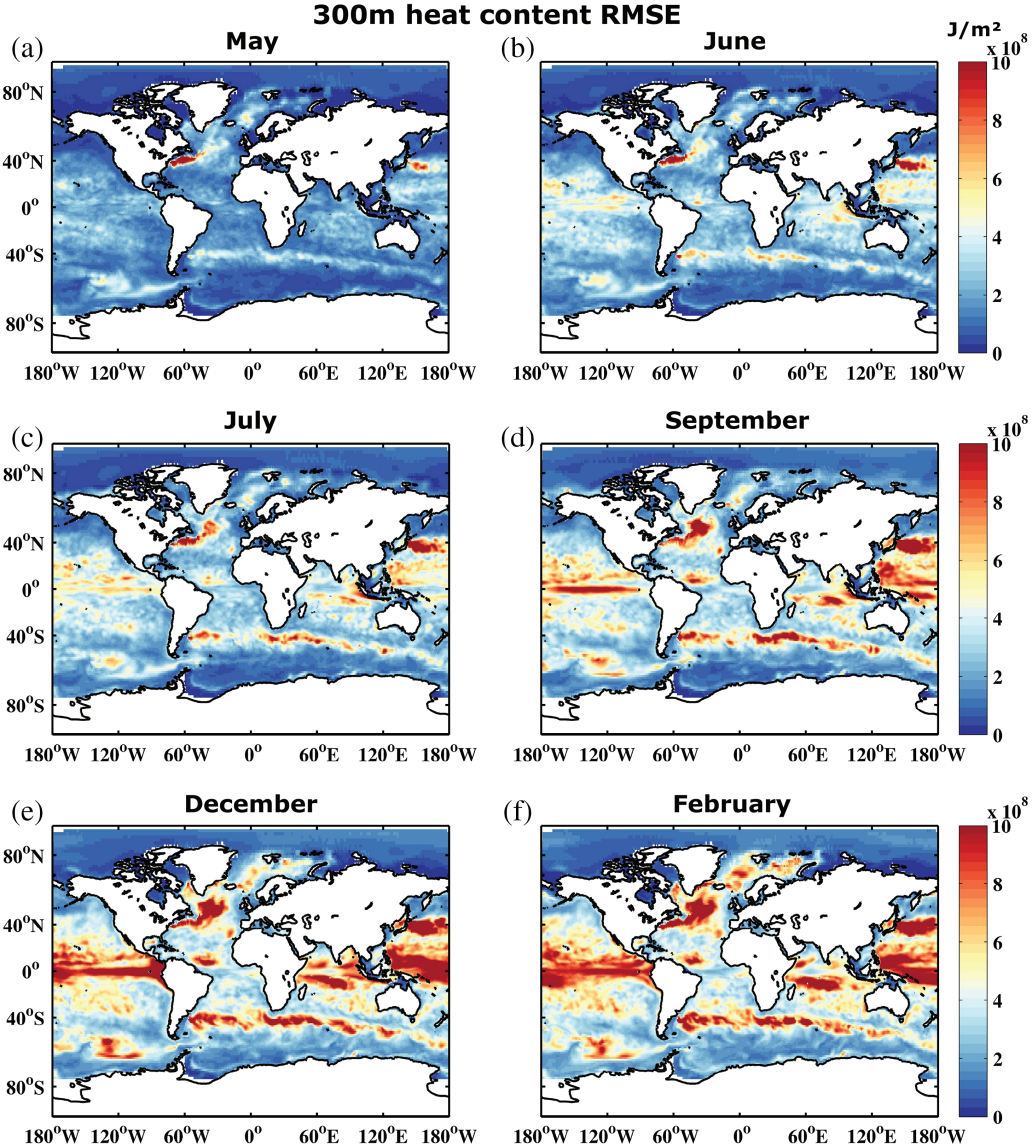
As Figure 2, but for upper 300 m ocean heat content (J m^−2^)

**Figure 5:**
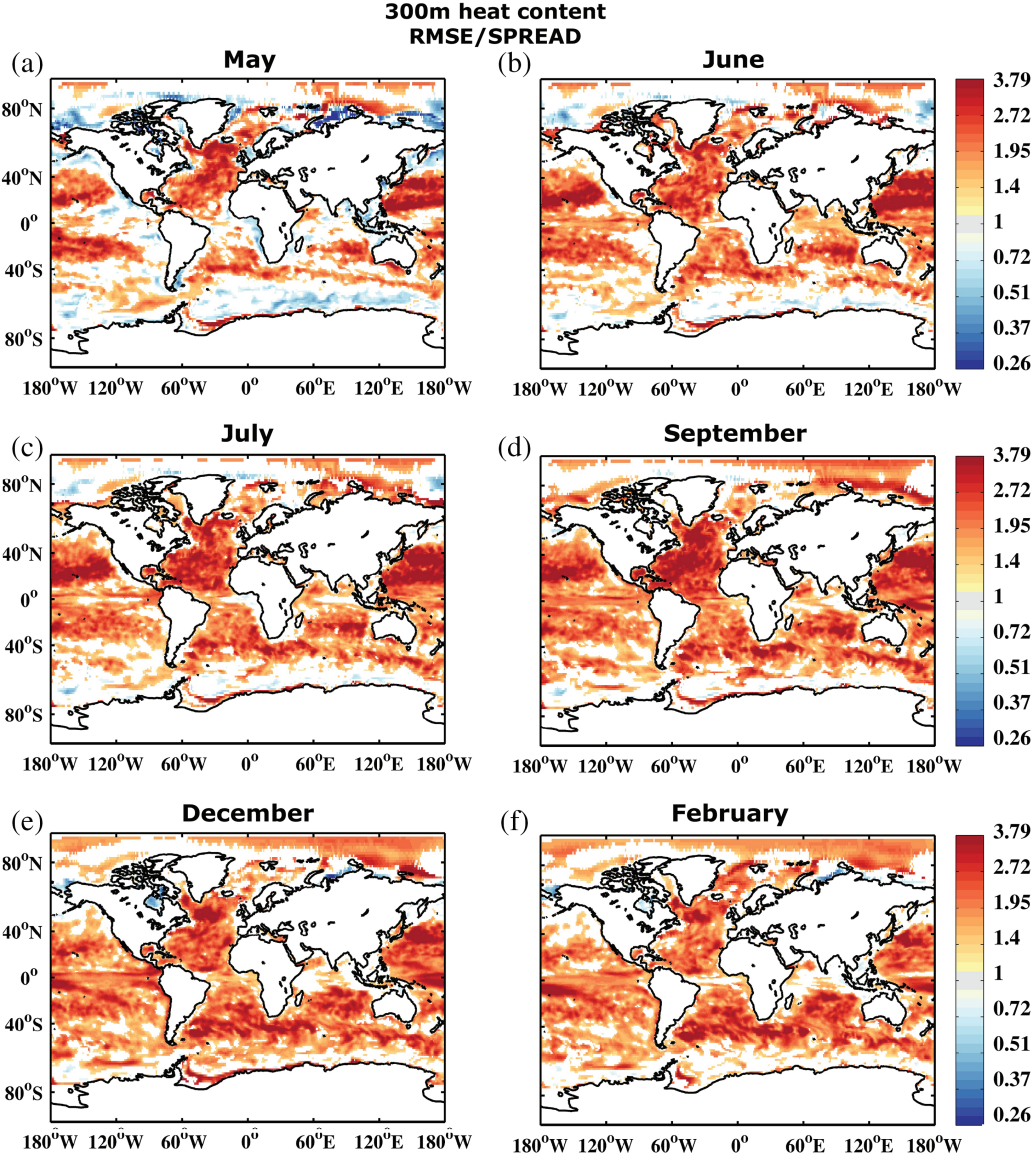
As Figure 3, but for upper 300 m ocean heat content. Note the nonlinear colour scale

In addition to SST reliability assessment, Figures 4 and 5 show the RMSE and RMSE/SPREAD ratio for integrated upper 300 m heat content. In contrast to SST, heat content does not exhibit a strong seasonal signal, either in SPREAD or RMSE. The impact of the seasonal surface forcing is reduced when vertically integrated quantities are considered. Error grows in regions where there is large variability throughout the entire upper ocean, especially along the western boundary currents and their extensions, in the Southern Ocean, and in the Tropics. The underdispersive nature of the ensemble becomes even more apparent for heat content (cf. Figures 3 and 5). Similar arguments as for SST hold, also for the slight overdispersion observed in some regions during the first forecast month.

As a comparison to the SST and upper‐ocean 300 m heat content RMSE of REF, we have also computed the RMSE of a climatological forecast (i.e. using monthly climatologies of ORAS4 as forecasts) and a persistence forecast (i.e. using April anomalies of ORAS4 for each of the ten forecast months). For SST the results suggest that REF performs better than climatology especially in the Tropics throughout the forecast but also in large areas of the midlatitudes until at least July (supporting material, Figures S1 and S3). REF performs worse than climatology especially in regions of strong model biases such as the North Atlantic, the Kuroshio, and parts of the high latitudes. Persistence provides a better forecast than climatology in most areas during the first two months, but generally performs worse than REF in most regions for all ten months (supporting material, Figures S2 and S4). This is due to the fact that SST anomalies generally last only a few weeks to months and the seasonal forecast model is able to predict this persistence as well. Similar to the climatological forecast, persistence has a reduced error compared to REF in regions of strong model biases. Results for upper‐ocean 300 m heat content are comparable to those for SST, except that the persistence forecast tends to perform better in the midlatitudes for slightly longer as anomalies in the subsurface can survive for longer (supporting material, Figures S5 and S6). However, climatology remains a better forecast than persistence during the last three months. In summary, REF outperforms both statistical forecasts with respect to heat content in most regions, especially during the last few months of the forecasts.

We would refer the reader to the supporting material for a more detailed discussion of differences in skill between persistence and climatological forecasts as basis of comparison for the dynamical forecasts. However, for the remainder of this article, we will keep climatology as basis of comparison, since it outperforms persistence in most instances.

### 3.2 Probablistic forecast skill

**Figure 6:**
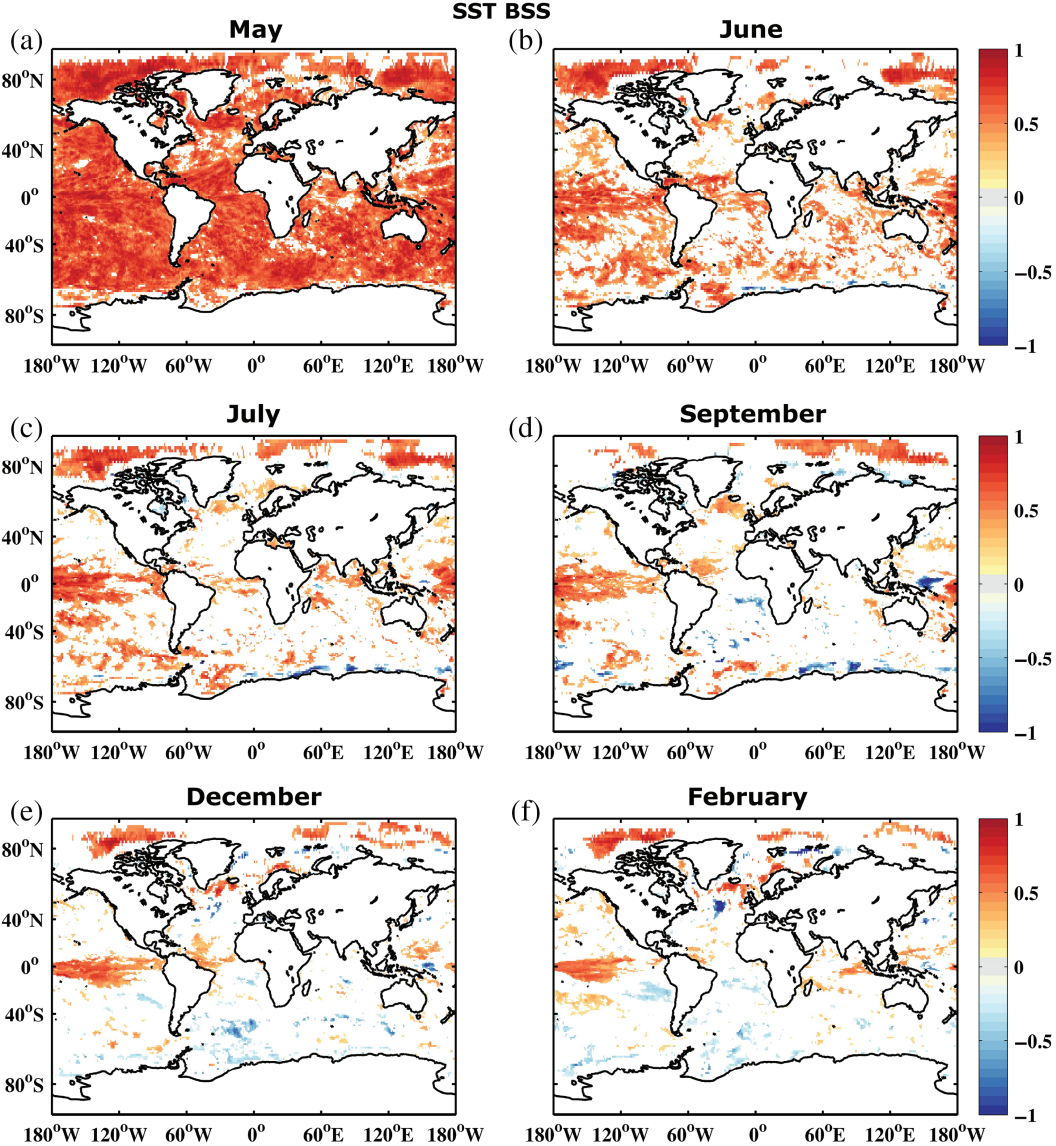
Brier skill score for sea surface temperature of the reference forecast REF compared to ORAS4 1° reanalysis for start dates 1981–2010 and months (a) May, (b) June, (c) July, (d) September, (e) December and (f) February. Areas shown are only those which are significantly different from 0 at 95% confidence according to a 1,000 sample bootstrapping with replacement

Figure 6 shows the BSS for SST referenced to ORAS4. The figure suggests that there is considerable predictive skill in the first month, which is related to the slower ocean time‐scales (compared to the atmosphere) and the fact that a persistence model on this time‐scale is already quite useful (e.g. (Barnston *et al.*, 2012)). Skill disappears quickly in the western boundaries, in large parts of the Southern Ocean and the central, midlatitude basins. However, in the Tropics, especially in the central Pacific, significant skill is maintained (i.e. positive BSS) until Feburary (month 10). These long‐term predictive modes are most likely related to low‐frequency tropical variability patterns such as ENSO. As mentioned before, there might be additional information gained from annual or multi‐annual forecasts when looking at longer averaging periods, for example annual means instead of monthly means. Analysis of annual anomalies might provide substantial skill in regions such as the North Atlantic (Zanna, 2012; Huddart *et al.*, 2016), or for atmospheric variables such as Sahel summer rainfall (Sheen *et al.*, 2017; O'Reilly *et al.*, 2017). As an indicator, the monthly means in our forecasts show some predictive skill in and northwards of the North Atlantic subpolar gyre on the ten‐month time‐scale. There is also some skill throughout most of the forecast in the tropical Atlantic.

A noteable drawback of the ECMWF seasonal forecasting system here is that it actually exhibits areas of negative skill, mostly in some regions of the Southern Ocean, from month 5 onwards. Negative skill means that the system performs worse than a system based on the mean climatology. This suggests model biases in the forecast distribution when compared to the climatology. While these areas are not exceedingly large, their patterns are relatively robust in time and location which reduces the probability of a simple sampling problem.

## 4 OBSERVATIONAL UNCERTAINTY

To investigate the sensitivity of the forecast verification to the choice of the reference reanalysis, we compare the diagnostics of the previous sections with similar diagnostics referenced to the ECMWF 1/4° reanalysis ORAP5. This comparison provides a lower bound on the impact of observational uncertainty, as the two reanalysis products do not sample the whole spectrum of ocean reanalyses. They both use to a large extent the same system that is also used for the seasonal forecast, are constrained by and assimilate similar data, and mainly differ in the horizontal resolution of the ocean model (1° compared to 1/4°), as well as vertical levels (42 compared to 75) and resolution‐dependent parametrization adjustments. It should be noted that the forecast model and both reanalysis models are based on the same ECMWF system.

### 4.1 Forecast error and reliability

**Figure 7:**
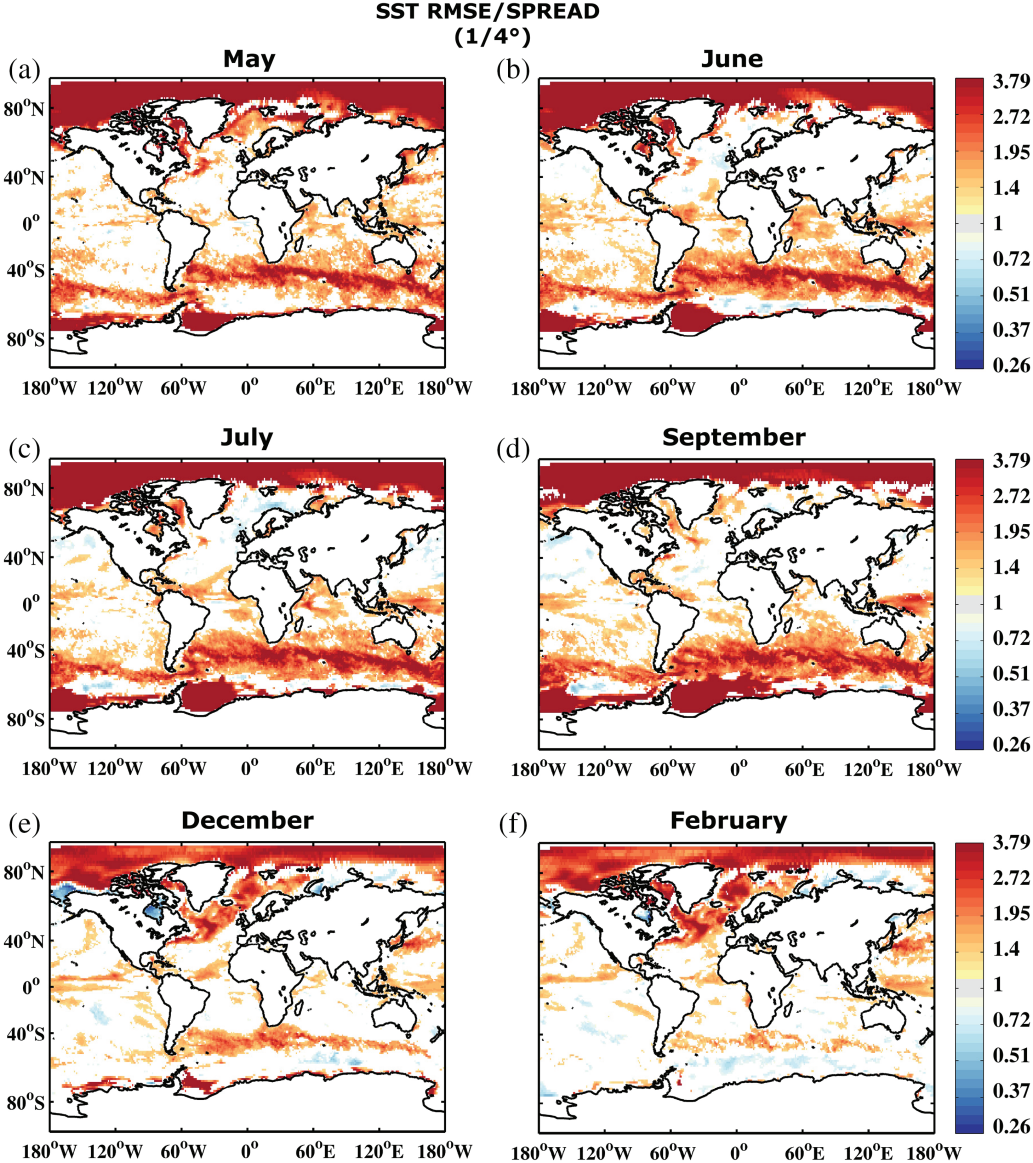
As Figure 3, but referenced to ORAP5 (1/4)° reanalysis. Note the nonlinear colour scale

Figure 7 shows the same RMSE/SPREAD ratio for SST as Figure 3 but referenced to the 1/4° ORAP5 rather than 1° ORAS4 reanalysis. The largest differences between Figures 3 and 7 are visible in the first month. While Figure 3a suggests that the system is overdispersive in May, Figure 7a suggests the exact opposite. Compared to ORAP5 the seasonal forecast is highly underdispersive in the first month, especially in the Southern Ocean. This can partly be explained by the fact that the forecast is initialized with the ORAS4 reanalysis spread (section 3.1). While that leads to too large a spread when compared to ORAS4, the comparison to ORAP5 suggests that the initial condition spread might actually not be large enough to capture the error growth. This result has strong implications for the design of initial condition perturbations. Figure 3 suggests that the initial condition spread might have been too large and the underdispersion in later months is mainly due to the model's inability to generate sufficient variability. However, Figure 7 suggests that the initial spread is already too low, especially in the Southern Ocean. The underdispersion is probably linked to underestimated initial uncertainty and the simulation of insufficient growth rates of the applied initial condition perturbations.

The general conclusion that REF referenced to ORAP5 rather than ORAS4 shows stronger underdispersion in the Southern Ocean remains true at least until September (month 5). After that, the two diagnostics become more similar, with some stronger underdispersion visible in the North Atlantic for the ORAP5 reanalysis reference. In the Tropics the two diagnostics are much more similar throughout the entire forecast range. In general, however, the ORAP5 reanalysis suggests slightly stronger underdispersion everywhere (i.e. larger RMSE).

This difference in midlatitude RMSE when referenced to ORAP5 instead of ORAS4 is also not just a consequence of interpolation of more highly resolved data to a lower‐resolution grid. Spatial smoothing of the monthly SST fields (supporting material, Figure S7) reveals that the difference remains large in the midlatitudes, which suggests that it originates only to some degree from the interpolation of an eddy‐permitting reanalysis to a grid that does not resolve eddies. Instead, the observed differences are to a large extent an effect of the eddy–mean flow interaction (or absence thereof) in the reanalysis model that causes the differences between ORAP5 and ORAS4 (Juricke *et al.*s*(2017) give a discussion of resolution effects on interannual ocean variability in the two reanalysis).

Furthermore, the very strong reanalysis dependence in the high latitudes can be attributed to two main reasons. For one, the seasonal forecasting model used for the ensemble forecasts in this study does not dynamically predict sea ice but uses sampling from previous years to generate sea ice conditions. Therefore sea ice does not evolve dynamically given the forcings by ocean and atmosphere. Diagnostics are likely to highlight differences in the high latitudes with strong diagnostic gradients to the midlatitudes as seen especially in Figure 7. The other reason is that the two reanalysis products also strongly differ in their sea ice treatment. While the model used for the ORAS4 reanalysis did not have a dynamical sea ice model (similar to the seasonal forecast model), ORAP5 does. Additionally, ORAP5 does assimilate sea ice concentration into the dynamical model for the first time in an ECMWF forecasting system. The sea ice and surface ocean state estimate in the high latitudes is therefore in better agreement with the ocean model state and the atmospheric forcing. It is allowed to vary dynamically. That is the reason why Figure 7 especially highlights the high latitudes as exceedingly underdispersive.

Finally, it should be noted that the choice of reference reanalysis does not affect the general conclusions of the RMSE comparison between REF, climatological and persistence forecasts in the previous section.

### 4.2 Probablistic forecast skill

**Figure 8:**
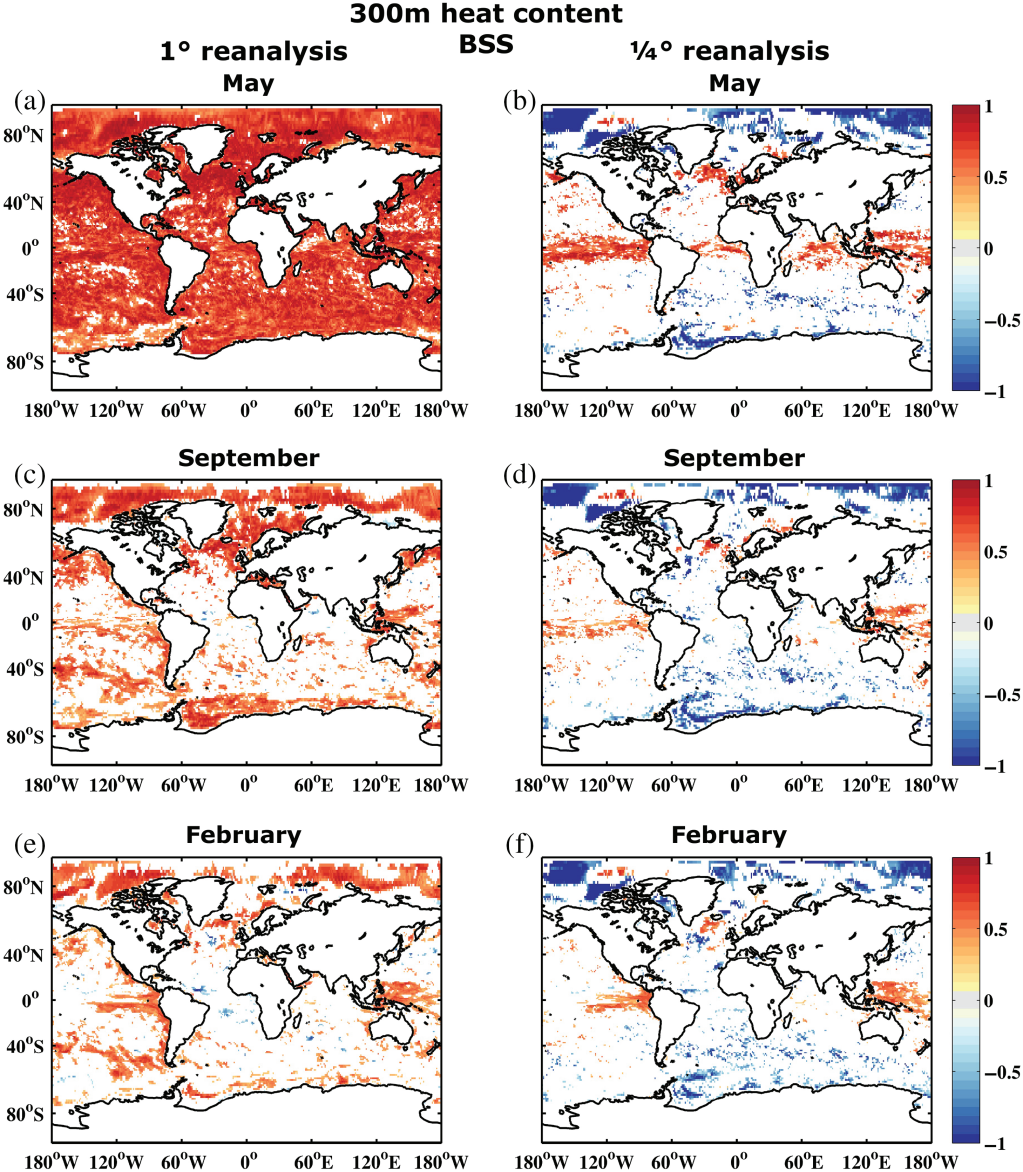
Brier skill score of upper 300 m ocean heat content of the reference forecast REF compared to (a, c, e) ORAS4 1° and (b, d, f) ORAP5 (1/4)° reanalysis for start dates 1981–2010 and months (a, b) May, (c, d) September, and (e, f) February. Areas shown are only those which are significantly different from 0 at 95% confidence according to a 1,000 sample bootstrapping with replacement

Figure 8 shows 300 m heat content BSS referenced to both the ORAS4 and ORAP5 reanalysis. Once again, the first month shows the strongest differences. While ORAS4 suggests predictive skill basically everywhere, this is reduced to skill only in the Tropics and some parts of the North Atlantic when referenced to ORAP5. For the high latitudes and some parts of the Southern Ocean, it actually switches from positive skill to negative skill. As the forecast is initialized with the ORAS4 reanalysis members, it is not surprising that it performs better in the first month when compared to the same ORAS4 reanalysis. However, the amplitude of the differences is considerable, especially since the switch between reanalysis products in the forecast verification process is between reanalyses that are technically still relatively close to each other.

The differences are also not limited to the first month, where they can mainly be explained by the initialization process. Throughout the entire forecast, referencing to ORAS4 suggests much higher skill than referencing to ORAP5, possibly with the exception of the Tropics. Here, the difference in resolution of the ocean reanalyses is not as critical as it is in the midlatitudes, where the change from 1° to 1/4° means a change from non‐eddy‐resolving to eddy‐permitting. Also, even with a 1° grid, the resolution is strongly meridionally refined in the Tropics to better simulate wave propagation, which makes the resolution between 1° and 1/4° grids more comparable in the Tropics. However, outside the Tropics, the differences in diagnosed skill are very large. So large in fact that, for large parts of the mid to high latitudes, ORAP5 claims that the forecast has as much or even less skill than climatology, while referencing to ORAS4 exhibits large areas of significant skill. For SST the two reference reanalyses provide much more similar results (not shown), although not for the first month where the ORAS4 reference provides much higher skill scores. Also, even for SST, ORAS4 is generally slightly more optimistic than ORAP5 throughout the entire forecast. In summary, this suggests that especially for subsurface fields – where reanalysis products are not well constrained by observations – the forecast verification strongly depends on the chosen reanalysis product. But even for better‐observed surface fields, it is crucial how the reanalysis model incorporates observational estimates for a given grid resolution to generate the reference data (Massonnet *et al.*, 2016).

## 5 MODEL UNCERTAINTY

### 5.1 Impact on forecast spread, error, and reliability

**Figure 9:**
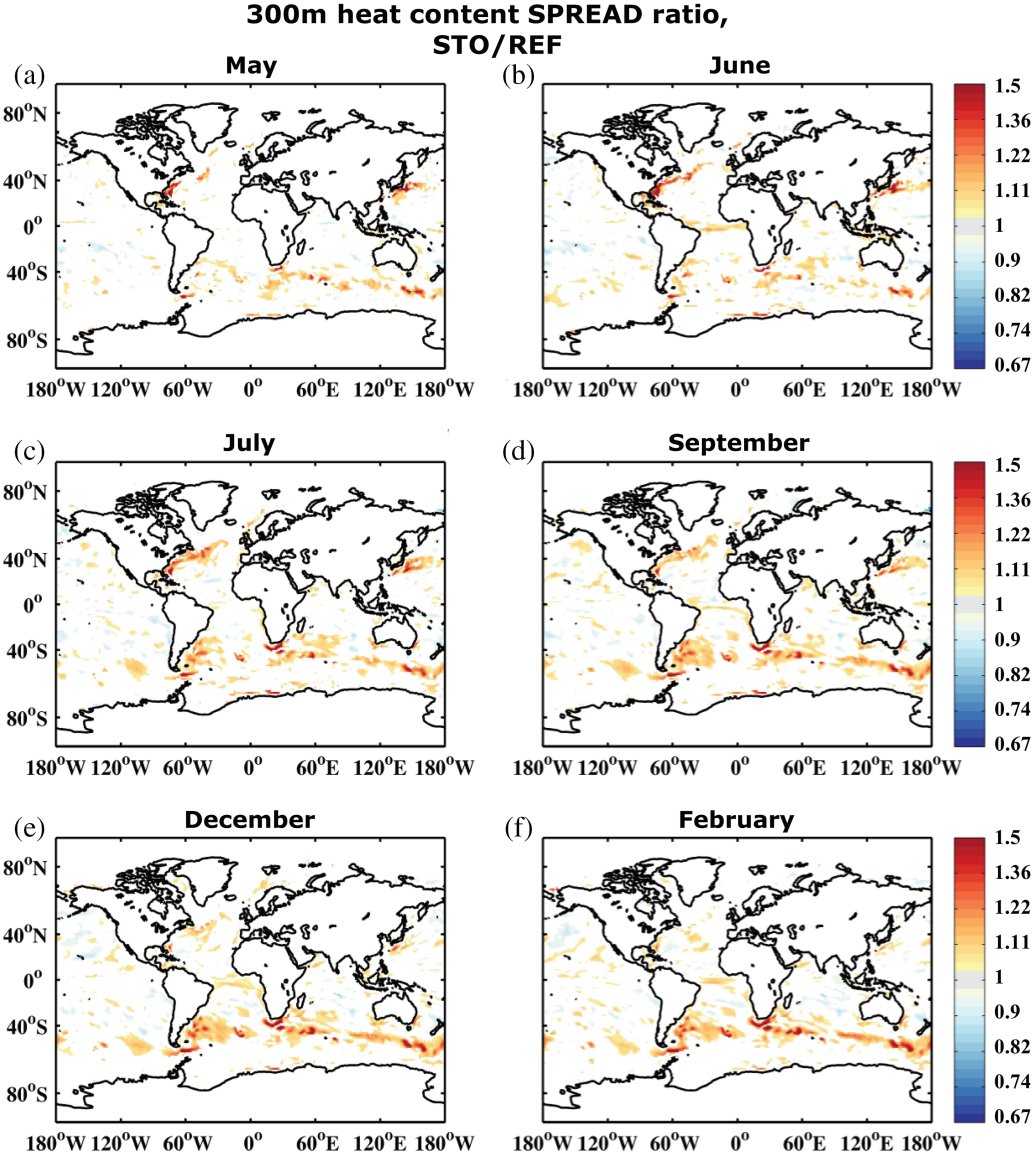
Ratio of upper 300 m ocean heat content ensemble spread between STO and REF averaged over the 30 start dates 1981–2010 for months (a) May, (b) June, (c) July, (d) September, (e) December and (f) February. Significant differences are shown only where the value of the upper quartile of the respective smaller spread (e.g. of REF) does not reach the value of the lower quartile of the respective larger spread (e.g. of STO), with quartiles generated by the 1,000 sample bootstrapping distributions (with replacement) for STO and REF. Similar significance estimates are found when considering only those differences where STO lies outside the 90% confidence interval of REF

To assess how model uncertainty affects forecast skill, we have run the two forecast sets REF and STO as described in section 2. The stochastic perturbations to the three parametrization schemes were mainly introduced to improve reliability. Introducing stochastic perturbations may lead to increased spread which evolves dynamically during the forecast and reduces underdispersion. This has already been shown in the study by Andrejczuk *et al.*s*(2016) for eddy‐active regions and a forecast length of three months, using ocean SPPT to account for model uncertainty. Figure 9 shows the increase in 300 m heat content SPREAD (relative to REF) generated by STO, starting with July (month 3). The first two months do not show a very strong signal. It is clear that, in agreement with Andrejczuk *et al.*s*(2016), especially the western boundary currents (i.e. Gulf Stream and Kuroshio), the Southern Ocean, and to some extent also the tropical Atlantic show increased SPREAD for STO, by a magnitude of up to 40%. There is some seasonal dependence of the signal similar to Figure 1, and the changes are relatively localized. In general, the increased SPREAD leads to a better calibrated system, i.e. reduced underdispersion (cf. Figure 5). The 300 m heat content RMSE is also on average slightly reduced (not shown) but the impact is more noisy and therefore less clear.

While 300 m heat content SPREAD has a strong ocean dynamics and diffusion component, SST SPREAD at this ocean model resolution is largely governed by atmospheric variability (Andrejczuk *et al.*, 2016). This is why the impact of the stochastic schemes on SST SPREAD is much less pronounced (not shown), although it shows a general increase in SPREAD in the Southern Ocean for September to December (months 5 to 8). Furthermore, SST RMSE is reduced during the last three months of the forecast, which will be discussed below in the context of SST BSS.

### 5.2 Impact on probablistic forecast skill

#### 5.2.1 Ocean

**Figure 10:**
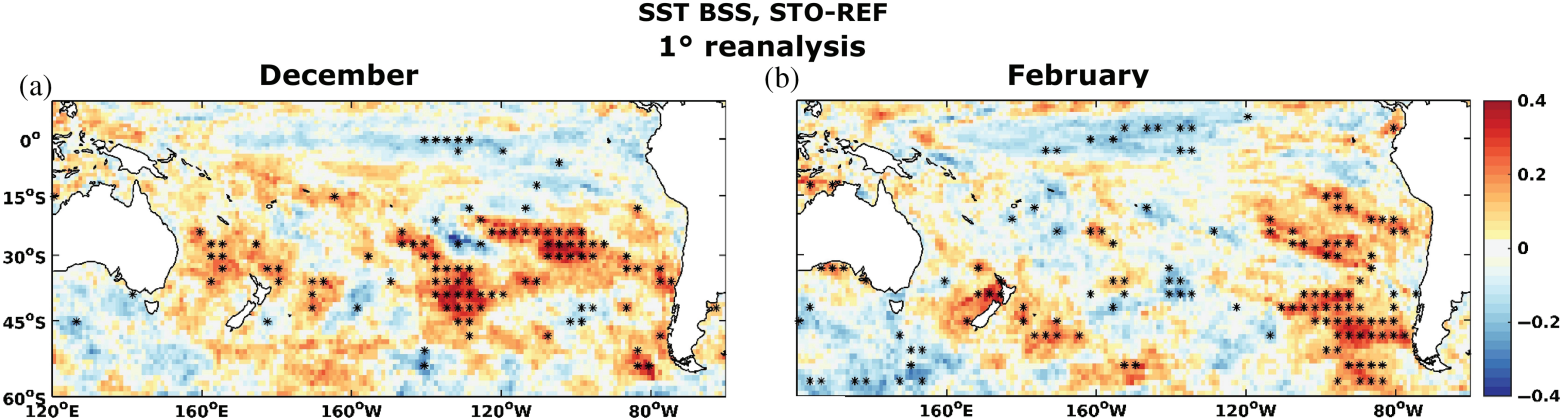
Difference in sea surface temperature Brier skill score between STO and REF for start dates 1981–2010 and months a) December and (b) February, referenced to ORAS4 1° reanalysis. Differences referenced to the ORAP5 (1/4)° reanalysis are similar. Stippled areas indicate significant differences according to where the value of the upper quartile of the respective lower score (e.g. of REF) does not reach the value of the lower quartile of the respective higher score (e.g. of STO), with quartiles generated by the 1,000 sample bootstrapping distributions (with replacement) for STO and REF. Similar significance estimates are found when considering only those differences where STO lies outside the 90% confidence interval of REF. Stippling is applied to a subset of grid points

Similar to Andrejczuk *et al.*s*(2016), the impact of incorporating model uncertainty estimates does not affect probabilistic skill scores significantly on the three‐month time‐scale. However, on longer time‐scales beyond November (month 7), the impact is also visible in the BSS especially for SST, in the midlatitude Pacific regions and in the South Atlantic, with largest significant changes in the South Pacific (Figure 10; also supporting material for global maps, Figure S8). Here SST shows a stronger signal than heat content. Analysis of SST RMSE difference between STO and REF suggests that the improvements are related to reduced RMSE in those regions (Figure S8). Additionally, while SPREAD changes for SST are less consistent than for heat content, SST SPREAD of STO is increased compared to REF especially in the southern midlatitude Pacific, the South Atlantic, and south of South America and Africa during the last three months (not shown). This is most dominant in February. However, it is difficult to assess the effect of the SPREAD increase, since this alone does not necessarily improve skill scores, especially when the focus is not on extremes. But strong changes in SPREAD in similar regions as the BSS improvements suggest at least some connection between the two. Our conclusion is that the BSS improvements due to STO are related to a combination of an improved ensemble mean (i.e. improved mean forecast trajectory) as well as improved reliability and dynamically evolving SPREAD.

Comparing the regions where the improvements are visible with the sensitivity studies carried out by Juricke *et al.*s*(2017), we can identify the schemes that are most likely responsible for the improvements. The increase in SPREAD is mostly visible in the vertically integrated fields of the western boundary currents and the Southern Ocean, with some imprints of SST SPREAD increase south of South America and Africa. These are areas where especially the perturbations to the GM scheme have a large impact. However the changes in the BSS are most pronounced in surface fields of the Tropics and Subtropics, where the perturbations to the TKE scheme are much more important. These perturbations also affect mixed‐layer depth and through this the intensity of surface coupling.

Interestingly, the improvements due to the stochastic schemes are robust, independent of the reference reanalysis used to calculate the BSS (not shown). While the absolute value of the BSS differs depending on which reanalysis is used, there is hardly any difference in the relative comparison of the skill score between STO and REF. Therefore, it is still possible to objectively investigate the changes caused by new model developments, at least in this case more or less independently of the references used for the verification.

Finally, we would like to note that, while SPREAD increase for upper‐ocean 300 m heat content is large in the Southern Ocean and western boundary currents (Figure 9), it does not reflect strongly in the BSS, either for heat content or SSTs. Since atmospheric variability in the Southern Ocean is a dominating factor for total surface variability on the seasonal time‐scale, the increase in SPREAD does not impact surface skill scores except for the regions south of South America and Africa. We speculate that the increase in heat content SPREAD on the ten‐month time‐scale is not yet sufficient to considerably improve or even change probabilistic skill scores in large parts of the Southern Ocean, although we do see some positive and negative significant changes in SST BSS in the southern Indian Ocean (supporting material, Figure S8). These differences will most likely become larger with increased forecast length, as the increase in upper 300 m ocean heat content SPREAD needs to compete with strong atmospheric variability to ultimately affect SST forecasts. Also, it should be noted once more that increased SPREAD does not necessarily improve skill scores, especially when the focus is on the probabilities of being above or below the median. Increased SPREAD will most likely have a stronger impact on extreme events, the assessment of which is outside the scope of this paper.

#### 5.2.2 Atmosphere

**Figure 11:**
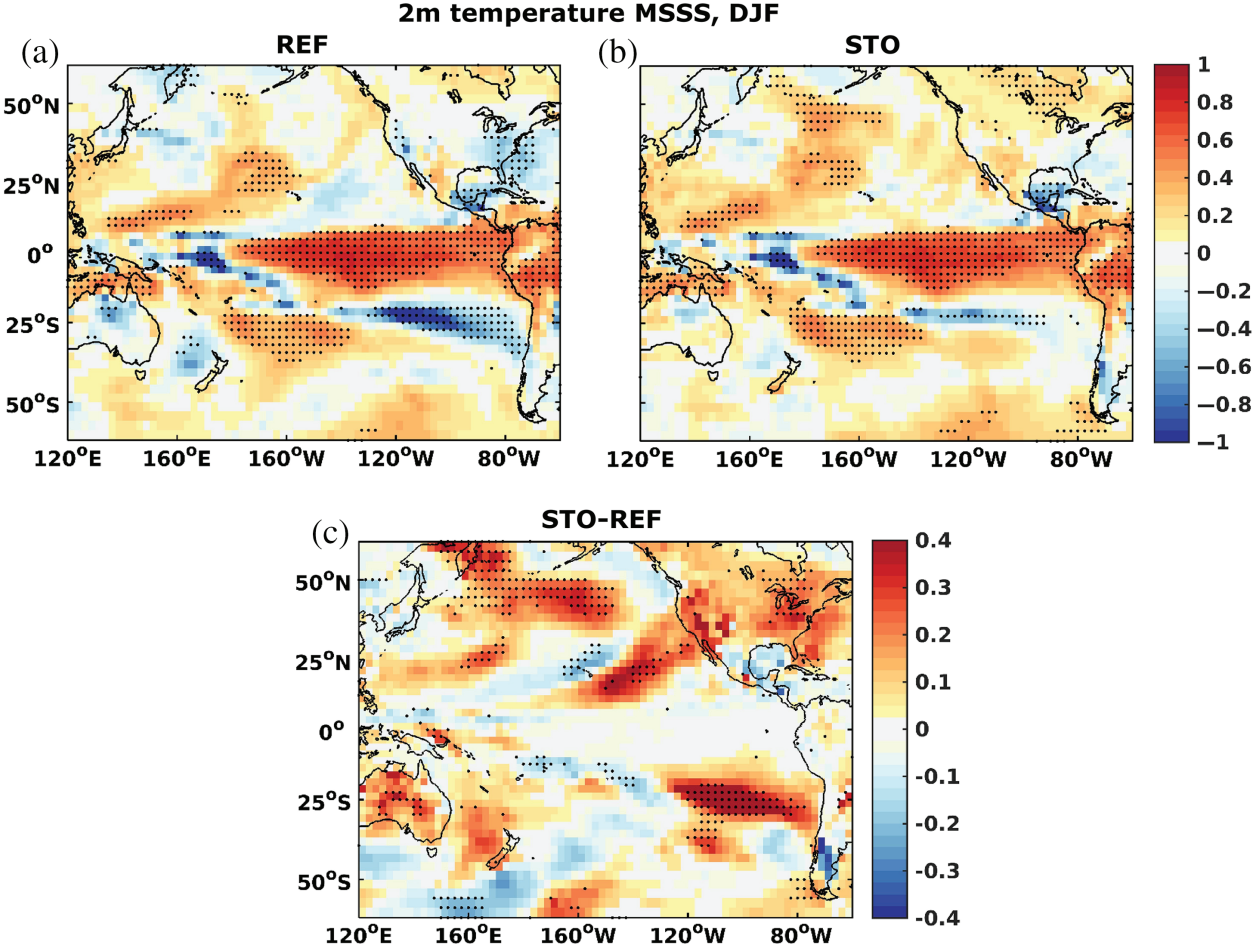
Air temperature at 2 m mean squared skill score of (a) REF, (b) STO and (c) the difference between STO and REF for start dates 1981–2010 and the averaged forecast months December to February, referenced to ERA‐Interim. Stippled areas in (a) and (b) indicate where the score for the two ensembles is significantly different from 0 at 95% confidence, according to 1,000 sample bootstrapping with replacement. Stippled areas in (c) indicate where the scores of the two ensembles differ from each other at 95% confidence, according to a 1,000 sample bootstrapping with replacement of the difference STO‐REF

Figure 11 shows that, aside from the ocean SST, 2 m air temperature skill also profits from the stochastic perturbations. This is shown here for the mean squared skill score (MSSS) which is an ensemble mean score. The score was chosen because it shows the clearest, smoothest signal, though similar results hold for probabilistic skill scores such as the BSS, if with somewhat reduced amplitude (not shown). Figure 11 shows large improvements in MSSS for the entire last season, i.e. DJF (months 8–10), with a focus on the Pacific where impacts of STO are largest. These improvements are not confined to the oceans, where Figure 10 already showed the positive impact in SSTs, but extend over the continents as well. Especially over North America, negative skill disappears while positive skill emerges, mostly over Canada. There appears to be a general pathway of improvements radiating from the western tropical/subtropical Pacific to the midlatitudes of the Americas which is more pronounced for the Northern Hemisphere (Figure 11c). However, there is also a slight reduction of skill in the tropical central Pacific (also Figure 10) which might be related to increased biases (i.e. shoaling) in both mixed‐layer and turbocline depth (not shown). The latter is a rather model‐dependent diagnostic variable. However, for both reanalysis products and the forecasts, it is consistently defined as the depth where the vertical eddy diffusivity falls below a certain threshold (here 5 cm s^−2^). This is directly affected by the vertical mixing parametrization (see NEMO documentation within the model code) and is a measure of vertical mixing activity in the upper ocean. These biases could potentially be improved by a slight retuning of the parameter regime. However, probabilistic skill scores of the mixed‐layer and turbocline depth are not affected, although we do see a slight decrease in probabilistic skill of the tropical 300 m ocean heat content. This decrease in skill is mostly confined to the central and eastern tropical Pacific and does not seem to affect the Subtropics and midlatidues, where the increase in skill is observed in Figures 10 and 11. On the other hand, the skill improvement is most likely due to an improved propagation of signals from the (western) tropical Pacific to the midlatitudes caused by a better representation of upper‐ocean conditions and hence surface coupling with the ocean. This in turn is most likely achieved by the stochastic TKE perturbations. We do not see any consistent impact on mixed‐layer depth biases in these regions but, as already mentioned, STO leads to a reduced SST RMSE, i.e. a better ensemble mean forecast (Figure S8). Similarly, the slight decrease in skill south of New Zealand and Australia, which is visible both in the ocean in Figure 10 and in the atmosphere in Figure 11, is related to an increase in SST RMSE (Figure S8). Finally, it should also be noted that, while we do see a slight decrease in local skill in the central tropical Pacific in Figure 10, the skill for none of the common spatially averaged El Niño indices is significantly reduced (not shown).

Aside from BSS and MSSS, other scores were also investigated for the atmosphere as part of the ECMWF diagnostics suite available for the seasonal forecasts, including the ignorance score and BSS for median, and upper and lower terciles, as well as the ensemble mean anomaly correlation (not shown). All these results agree with the results presented here. Furthermore, other atmospheric fields were analysed, such as precipitation and sea level pressure. While most of these were inconclusive (for example precipitation), the ones that showed a significant signal (for example sea level pressure) were related to the above‐mentioned improvements in the Subtropics and midlatitudes or the slight decrease in skill in the tropical Pacific.

## 6 SUMMARY AND CONCLUSIONS

In this study a comprehensive set of seasonal to annual forecasts was analysed, focusing on the forecast skill of ocean variables on a ten‐month time‐scale. An updated version of the ECMWF seasonal forecasting model System 4 was used with an ocean model resolution of 1°. Forecasts were initialized in May and carried out for ten months, with start years 1981–2010 and an ensemble size of 20 members.

In the Tropics, results show that the dynamical ocean forecasts exhibit up to ten‐month SST forecast skill for the probabilistic Brier skill score and outperform simple climatological and persistence forecasts in terms of root mean squared error, especially in the central Pacific. Almost everywhere else, the skill drops quickly to zero, with the exception of the far North Atlantic. Similar results hold for upper 300 m heat content, although the skill is retained slightly longer during the first few months (if referenced to the 1° ECMWF ocean reanalysis). Throughout the forecast the system remains underdispersive for ocean variables, particularly in turbulent regions such as the Southern Ocean. Especially sea surface temperature shows a strong seasonal signal in the ensemble spread and forecast error, which balances to a large degree for the reliability. Investigation of the impact of different start dates will be left for future studies.

A comparison of Brier skill scores and reliability referenced to two different reanalysis products, namely the default ECMWF 1° and the recent 1/4° reanalysis, revealed a strong reference data dependence, not just for subsurface (more scarcely assimilated) quantities but also for SSTs. Especially during the first month, slightly overdispersive regions for SST are highlighted as underdispersive when moving from the 1° to the newer 1/4° reanalysis. In line with this, Brier forecast skill drops significantly, especially for the 300 m heat content. Increased amplitude of underdispersive regions and generally reduced skill remains true throughout the forecast when choosing the 1/4° reanalysis as reference.

To account for model uncertainty, stochastic perturbations to three main mixing parametrizations (described by (Juricke *et al.*, 2017)) were introduced. The applied stochastic perturbations account for uncertain parameters and parametrized tendencies within these parametrizations. Another set of forecasts with the stochastic ocean schemes was initialized for the same dates and with the same ensemble size, and compared to the reference forecasts without stochastics in the ocean model.

The stochastic schemes improve the model reliability especially for 300 m heat content from forecast month 3 onwards. The Brier skill score was significantly improved for sea surface temperature in the subtropical and midlatitude Pacific during the last three months of the forecasts. These improvements were robust, independent of the reference dataset used to validate the model performance. They are a consequence of improved ensemble mean forecasts and increased ensemble spread, i.e. ensemble forecast distribution. Finally, forecast skill was also significantly improved for 2 m air temperature, leading to significant forecast skill over North America during the last three months of the forecast. This skill was absent in the reference simulation. The results showed that using stochastic schemes to represent model uncertainty can both improve model reliability and forecast skill scores. The former is mostly achieved by increasing model spread in dynamically active regions. The latter results from a better representation of both the ensemble mean as well as the probabilistic information contained in the distribution of the ensemble members.

It should be noted that the improvements due to incorporation of ocean model uncertainty discussed in this paper are based on the three stochastic schemes implemented (Juricke *et al.*, 2017). This is not an exhaustive set of model uncertainty estimates but only comprises some schemes with relatively large related uncertainties. Each scheme also acts on different time‐scales, from a couple of weeks to decades, and might have some seasonal dependence. Therefore, depending on the forecast length, initialization, and focus, some schemes might be more effective than others in improving forecast skill for different lead times. This is also the case for stochastic schemes implemented in other components of the forecasting system. Furthermore, the already implemented schemes might develop even stronger impacts once the model resolution allows for the development of eddies and more chaotic, active behaviour through reduced viscosity. The non‐eddy‐resolving resolution of the current forecasts leads to a much more linear behaviour of the ocean model than is observed in the real, eddying ocean (Andrejczuk *et al.*, 2016; Juricke *et al.*, 2017). Increases in resolution may impact the schemes' effect on the climatic mean state and variability patterns differently. Similar to a deterministic model that incorporates a new scheme, retuning of the model might become necessary, which is here already apparent in the case of a slight bias increase (i.e. shoaling) for the tropical Pacific turbocline and mixed‐layer depth.

This study shows that there is still a lot of forecast skill to be gained by improvements in the model set‐up, not only from general model development, systematic error reduction (e.g. (Zadra *et al.*, 2018)) and increase in resolution (e.g. (MacLachlan *et al.*, 2015)), but also from adequately accounting for forecast model uncertainty (Leutbecher *et al.*, 2017). However, in the verification process, it should always be kept in mind that especially for the (subsurface) ocean, it is difficult to decide upon the one true state with which the model should be compared (e.g. (Karspeck *et al.*, 2017; Balmaseda *et al.*, 2015)). This is why observational uncertainty needs to be accounted for as well, also in the development of new diagnostics (Massonnet *et al.*, 2016; Bellprat *et al.*, 2017; Ferro, 2017). However, for this study, while absolute values of skill were dependent on the reference reanalysis, the forecast improvements gained by applying stochastic methods for model uncertainty estimation were more or less independent of that, providing robust indicators of skill increase.

## ACKNOWLEDGEMENTS

The seasonal forecasts were carried out with ECMWF supercomputing resources. This work was supported by the UK NERC Grants NE/K013548/1 and NE/J00586X/1. SJ and TNP were supported under the European Research Council Grant PESM 291406. DM and AW were supported by the FP7 project SPECS (grant agreement number 308378). With this paper SJ is contributing to the project M3 of the Collaborative Research Centre TRR 181 "Energy Transfer in Atmosphere and Ocean" funded by the German Research Foundation. We thank two anonymous reviewers and the associate editor for their helpful and constructive comments, improving and clarifying the final version of the paper.

## Supporting information


**Figure S1.** Root mean square error of sea surface temperature (*K*) between the ORAS4 1° reanalysis and its climatological forecast (i.e. mean squared ORAS4 anomalies) for years 1981‐2010 and months a) May, b) June, c) July, d) September, e) December and f) February.
**Figure S2.** Root mean square error of sea surface temperature (*K*) between the ORAS4 1° reanalysis and its presistence forecast (i.e. using the respective April anomaly for all ten forecast months) for years 1981‐2010 and months a) May, b) June, c) July, d) September, e) December and f) February.
**Figure S3.** Difference in root mean square error of sea surface temperature (*K*) between REF (Figure 1) and the climatological forecast (Figure S1) for the ORAS4 1° reanalysis for years 1981‐2010 and months a) May, b) June, c) July, d) September, e) December and f) February. Blue shading means REF has a lower RMSE, while red means the climatology of ORAS4 provides a better forecast. Climatology is more accurate than REF mostly in the North Atlantic and along the Kuroshio (from June onwards) due to large model biases in these regions.
**Figure S4.** Same as Figure S3 but for the difference between REF and the per‐sistence forecast (Figure S2). Blue shading means REF has a lower RMSE, while red means that persistence provides a better forecast. While persistence is a better forecast in large areas of the mid latitudes during the first two months, climatology is more accurate thereafter, as it compares in most areas more favorable to REF for July{February. Persistence is more accurate than REF mostly in the North Atlantic and also the Kuroshio due to large model biases in these regions.
**Figure S5.** Same as Figure S3 but for upper 300m ocean heat content (*J*/*m* ^2^). Compared to sea surface temperature in Figure S3 REF remains the more skilful forecast for much longer. Climatology is more accurate than REF mostly in the North Atlantic and along the Kuroshio (from September onwards) due to large model biases in these regions.
**Figure S6.** Same as Figure S4 but for upper 300m ocean heat content (*J*/*m* ^2^). Although persistence of heat content lasts a little longer than for sea surface temperature (compare RMSE difference progression through the year with Figure S4) the forecast is nearly everywhere less skilful than REF already after one month, and less skilful than the climatology for large areas from December onwards. Persistence is more accurate than REF mostly in the North Atlantic and also the Kuroshio (from July onwards) due to large model biases in these regions.
**Figure S7.** Effects of spatial smoothing on sea surface temperature root mean square error difference between REF verified against ORAP5 (1/4)° reanalysis and REF verified against OARS4 1° reanalysis (RMSE_ORAP5_ – RMSE_ORAS4_) for June of years 1981‐2010: a) no smoothing; b) smoothing filter applied once; c) smoothing filter applied twice. The smoothing filter uses a nine‐point stencil to compute a weighted mean. The central grid point has weight 1, the four direct neighbours have weight 0.5 and the diagonal neighbours have weight 0.3. Smoothing is performed on the REF grid for the SST fields from REF, ORAS4, and ORAP5. In the mid latitudes the smoothing operates approximately over the surrounding 100km, applying the smoothing twice increases this to around 200km. In the tropics this distance is smaller because of the telescoping of the grid. It is also smaller in the polar regions. Generally, the RMSE estimate of REF is larger when using ORAP5 as reference, signified by the red shading. As the smoothing is increased the difference between the error estimates for the two reanalyses is reduced but remains substantial in the mid latitudes. This implies that the difference does only to some degree originate from the interpolation of an eddy permitting reanalysis to a grid that does not resolve eddies.
**Figure S8.** Difference in sea surface temperature (top) Brier skill score as well as (bottom) root mean square error between STO and REF for start dates 1981‐2010 and months a) and c) December and b) and d) February, referenced to ORAS4 1° reanalysis. Stippled areas for BSS indicate significant differences according to where the value of the upper quartile of the respective lower score (e.g. of REF) does not reach the value of the lower quartile of the respective higher score (e.g. of STO), with quartiles generated by the 1000 sample bootstrapping distributions (with replacement) for STO and REF. Similar significance estimates are found when considering only those differences where STO lies outside the 90% confidence interval of REF. Areas of significant BSS increase (decrease) in STO, i.e. stippled red shading (blue shading) in a) and b), coincide with those areas where the RMSE for STO is reduced (increased) compared to REF, i.e. blue shading (red shading) in c) and d). This indicates that significant changes in BSS are to some extent caused by the changes in RMSE.

## References

[qj3394-bib-0001] Alves OJ , Balmaseda MA , Anderson DLT , Stockdale T . 2004 Sensitivity of dynamical seasonal forecasts to ocean initial conditions. Quarterly Journal of the Royal Meteorological Society 130: 597: 647–667, 10.1256/qj.03.25.

[qj3394-bib-0002] Andrejczuk M , Cooper FC , Juricke S , Palmer TN , Weisheimer A , Zanna L . 2016 Oceanic stochastic parameterizations in a seasonal forecast system. Monthly Weather Review 144: 5: 1867–1875, 10.1175/MWR-D-15-0245.1.

[qj3394-bib-0003] Arribas A , Glover M , Maidens A , Peterson K , Gordon M , MacLachlan C , Graham R , Fereday D , Camp J , Scaife AA , Xavier P , McLean P , Colman A , Cusack S . 2011 The GloSea4 ensemble prediction system for seasonal forecasting. Monthly Weather Review 139: 6: 1891–1910, 10.1175/2010MWR3615.1.

[qj3394-bib-0004] Balmaseda MA , Anderson DLT . 2009 Impact of initialization strategies and observations on seasonal forecast skill. Geophysical Research Letters 36: 1: L01 701, 10.1029/2008GL035561.

[qj3394-bib-0005] Balmaseda MA , Alves OJ , Arribas A , Awaji T , Behringer DW , Ferry N , Fujii Y , Lee T , Rienecker M , Rosati T , Stammer D . 2009 Ocean initialization for seasonal forecasts. Oceanography 22: 3: 154–159, 10.5670/oceanog.2009.73.

[qj3394-bib-0006] Balmaseda MA , Mogensen K , Weaver AT . 2013 Evaluation of the ECMWF ocean reanalysis system ORAS4. Quarterly Journal of the Royal Meteorological Society 139: 1132–1161.

[qj3394-bib-0007] Balmaseda MA , Hernandez F , Storto A , Palmer M , Alves OJ , Shi L , Smith G , Toyoda T , Valdivieso M , Barnier B , Behringer D , Boyer T , Chang YS , Chepurin G , Ferry N , Forget G , Fujii Y , Good S , Guinehut S , Haines K , Ishikawa Y , Keeley S , Köhl A , Lee T , Martin M , Masina S , Masuda S , Meyssignac B , Mogensen K , Parent L , Peterson K , Tang Y , Yin Y , Vernieres G , Wang X , Waters J , Wedd R , Wang O , Xue Y , Chevallier M , Lemieux JF , Dupont F , Kuragano T , Kamachi M , Awaji T , Caltabiano A , Wilmer‐Becker K , Gaillard F . 2015 The ocean reanalyses intercomparison project (ORA‐IP). Journal of Operational Oceanography 8: Suppl 1: s80–s97, 10.1080/1755876X.2015.1022329.

[qj3394-bib-0008] Barnston AG , Tippett MK , L'Heureux ML , Li S , DeWitt DG . 2012 Skill of real‐time seasonal ENSO model predictions during 2002‐11: Is our capability increasing?. Bulletin of the American Meteorological Society 93: 5: 631–651, 10.1175/BAMS-D-11-00111.1.

[qj3394-bib-0009] Batté L , Doblas‐Reyes FJ . 2015 Stochastic atmospheric perturbations in the ec‐earth3 global coupled model: impact of sppt on seasonal forecast quality. Climate Dynamics 45: 11: 3419–3439, 10.1007/s00382-015-2548-7.

[qj3394-bib-0010] Becker E , van den Dool H , Zhang Q . 2014 Predictability and forecast skill in NMME. Journal of Climate 27: 15: 5891–5906, 10.1175/JCLI-D-13-00597.1.

[qj3394-bib-0011] Bellprat O , Massonnet F , Siegert S , Prodhomme C , Macias‐Gómez D , Guemas V , Doblas‐Reyes F . 2017 Uncertainty propagation in observational references to climate model scales. Remote Sensing of the Environment 203: 101–108, 10.1016/j.rse.2017.06.034.

[qj3394-bib-0012] Berner J , Shutts GJ , Leutbecher M , Palmer TN . 2009 A spectral stochastic kinetic energy backscatter scheme and its impact on flow‐dependent predictability in the ECMWF ensemble prediction system. Journal of Atmospheric Sciences 66: 3: 603–626, 10.1175/2008JAS2677.1.

[qj3394-bib-0013] Berner J , Achatz U , Batté L , Bengtsson L , de la Cámara A , Christensen HM , Colangeli M , Coleman DRB , Crommelin D , Dolaptchiev SI , Franzke CLE , Friederichs P , Imkeller P , Järvinen H , Juricke S , Kitsios V , Lott F , Lucarini V , Mahajan S , Palmer TN , Penland C , Sakradzija M , von Storch JS , Weisheimer A , Weniger M , Williams PD , Yano JI . 2017 Stochastic parameterization: toward a new view of weather and climate models. Bulletin of the American Meteorological Society 98: 3: 565–588, 10.1175/BAMS-D-15-00268.1.

[qj3394-bib-0014] Brankart JM . 2013 Impact of uncertainties in the horizontal density gradient upon low resolution global ocean modelling. Ocean Modelling 66: 64–76.

[qj3394-bib-0015] Brankart JM , Candille G , Garnier F , Calone C , Melet A , Bouttier PA , Brasseur P , Verron J. . 2015 A generic approach to explicit simulation of uncertainty in the NEMO ocean model. Geoscientific Model Development Discussions 8: 1: 615–643, 10.5194/gmdd-8-615-2015.

[qj3394-bib-0016] Buizza R , Miller MJ , Palmer TN . 1999 Stochastic representation of model uncertainties in the ECMWF ensemble prediction system. Quarterly Journal of the Royal Meteorological Society 125: 2887–2908.

[qj3394-bib-0017] Cooper FC . 2017 Optimisation of an idealised primitive equation ocean model using stochastic parameterization. Ocean Modelling 113: 187–200, 10.1016/j.ocemod.2016.12.010.

[qj3394-bib-0018] Cooper FC , Zanna L . 2015 Optimisation of an idealised ocean model, stochastic parameterisation of sub‐grid eddies. Ocean Modelling 88: 38–53.

[qj3394-bib-0019] Dee DP , Uppala SM , Simmons AJ , Berrisford P , Poli P , Kobayashi S , Andrae U , Balmaseda MA , Balsamo G , Bechtold PBP , Beljaars ACM , van de Berg L , Bidlot J , Bormann N , Delsol C , Dragani R , Fuentes M , Geer AJ , Haimberger L , Healy SB , Hersbach H , Hólm EV , Isaksen L , Kållberg P , Köhler M , Matricardi M , McNally AP , Monge‐Sanz BM , Morcrette J‐J , Park BK , Peubey C , de Rosnay P , Tavolato C , Thépaut J‐N , Vitart F . 2011 The ERA‐Interim reanalysis: configuration and performance of the data assimilation system. Quarterly Journal of the Royal Meteorological Society 137: 656: 553–597.

[qj3394-bib-0020] Doblas‐Reyes FJ , García‐Serrano J , Lienert F , Biescas AP , Rodrigues LRL . 2013 Seasonal climate predictability and forecasting: status and prospects. Wiley Interdisciplinary Reviews: Climate Change 4: 4: 245–268, 10.1002/wcc.217.

[qj3394-bib-0021] Eade R , Smith D , Scaife AA , Wallace E , Dunstone N , Hermanson L , Robinson N . 2014 Do seasonal‐to‐decadal climate predictions underestimate the predictability of the real world?. Geophysical Research Letters 41: 15: 5620–5628, 10.1002/2014GL061146. 25821271 PMC4373130

[qj3394-bib-0022] Ferro CAT . 2017 Measuring forecast performance in the presence of observation error. Quarterly Journal of the Royal Meteorological Society 143: 2665–2676, 10.1002/qj.3115.

[qj3394-bib-0023] Grooms I . 2016 A Gaussian‐product stochastic Gent–McWilliams parameterization. Ocean Modelling 106: 27–43, 10.1016/j.ocemod.2016.09.005.

[qj3394-bib-0024] Ho CK , Hawkins E , Shaffrey L , Bröcker J , Hermanson L , Murphy JM , Smith DM , Eade R . 2013 Examining reliability of seasonal to decadal sea surface temperature forecasts: the role of ensemble dispersion. Geophysical Research Letters 40: 21: 5770–5775, 10.1002/2013GL057630.

[qj3394-bib-0025] Huddart B , Subramanian A , Zanna L , Palmer TN . 2016 Seasonal and decadal forecasts of Atlantic sea surface temperatures using a linear inverse model. Climate Dynamics 49: 5: 1833–1845, 10.1007/s00382-016-3375-1.

[qj3394-bib-0026] Juricke S , Jung T . 2014 Influence of stochastic sea ice parametrization on climate and the role of atmosphere–sea ice–ocean interaction. Philosophical Transactions of the Royal Society A 372: 20130283, 10.1098/rsta.2013.0283. PMC4024236 24842027

[qj3394-bib-0027] Juricke S , Lemke P , Timmermann R , Rackow T . 2013 Effects of stochastic ice strength perturbation on Arctic finite‐element sea ice modeling. Journal of Climate 26: 3785–3802.

[qj3394-bib-0028] Juricke S , Goessling HF , Jung T . 2015 Potential sea ice predictability and the role of stochastic sea ice strength perturbations. Geophysical Research Letters 41: 8396–8403.

[qj3394-bib-0029] Juricke S , Palmer TN , Zanna L . 2017 Stochastic subgrid‐scale ocean mixing: impacts on low‐frequency variability. Journal of Climate 30: 13: 4997–5019, 10.1175/JCLI-D-16-0539.1.

[qj3394-bib-0030] Karspeck AR , Stammer D , Köhl A , Danabasoglu G , Balmaseda MA , Smith DM , Fujii Y , Zhang S , Giese B , Tsujino H , Rosati A . 2017 Comparison of the Atlantic meridional overturning circulation between 1960 and 2007 in six ocean reanalysis products. Climate Dynamics 49: 3: 957–982, 10.1007/s00382-015-2787-7.

[qj3394-bib-0031] Kumar A , Peng P , Chen M . 2014 Is there a relationship between potential and actual skill?. Monthly Weather Review 142: 6: 2220–2227, 10.1175/MWR-D-13-00287.1.

[qj3394-bib-0032] Leutbecher M , Lock SJ , Ollinaho P , Lang STK , Balsamo G , Bechtold P , Bonavita M , Christensen HM , Diamantakis M , Dutra E , English S , Fisher M , Forbes RM , Goddard J , Haiden T , Hogan RJ , Juricke S , Lawrence H , MacLeod D , Magnusson L , Malardel S , Massart S , Sandu I , Smolarkiewicz PK , Subramanian A , Vitart F , Wedi N , Weisheimer A . 2017 Stochastic representations of model uncertainties at ECMWF: state of the art and future vision. Quarterly Journal of the Royal Meteorological Society 143: 2315–2339, 10.1002/qj.3094.

[qj3394-bib-0033] MacLachlan C , Arribas A , Peterson KA , Maidens A , Fereday D , Scaife AA , Gordon M , Vellinga M , Williams A , Comer RE , Camp J , Xavier P , Madec G. . 2015 Global seasonal forecast system version 5 (GloSea5): a high‐resolution seasonal forecast system. Quarterly Journal of the Royal Meteorological Society 141: 1072–1084, 10.1002/qj.2396.

[qj3394-bib-0034] MacLeod DA , Cloke HL , Pappenberger F , Weisheimer A . 2016 Improved seasonal prediction of the hot summer of 2003 over Europe through better representation of uncertainty in the land surface. Quarterly Journal of the Royal Meteorological Society 142: 79–90, 10.1002/qj.2631.

[qj3394-bib-0035] Madec G . 2008 NEMO ocean engine. Institute Pierre‐Simon Laplace: Paris. Note du Pôle de modélisation 27.

[qj3394-bib-0036] Martin M , Balmaseda M , Bertino L , Brasseur P , Brassington G , Cummings J , Fujii Y , Lea D , Lellouche JM , Mogensen K , Oke P , Smith G , Testut CE , Waagbø G , Waters J , Weaver A . 2015 Status and future of data assimilation in operational oceanography. Journal of Operational Oceanography 8: Suppl 1: s28–s48, 10.1080/1755876X.2015.1022055.

[qj3394-bib-0037] Massonnet F , Bellprat O , Guemas V , Doblas‐Reyes FJ . 2016 Using climate models to estimate the quality of global observational data sets. Science 354: 6311: 452–455, 10.1126/science.aaf6369. 27789838

[qj3394-bib-0038] Mogensen K , Balmaseda MA , Weaver AT . 2012 The NEMOVAR Ocean Data Assimilation System as Implemented in the ECMWF Ocean Analysis for System 4. ECMWF: Reading. Technical Memorandum 668.

[qj3394-bib-0039] Molteni F , Stockdale T , Balmaseda MA , Balsamo G , Buizza R , Ferranti L , Magnusson L , Mogensen K , Palmer TN , Vitart F . 2011 The New ECMWF Seasonal Forecast System (System 4). ECMWF: Reading. Technical Memorandum 656.

[qj3394-bib-0040] van Oldenborgh GJ , Balmaseda MA , Ferranti L , Stockdale TN , Anderson DLT . 2005 Evaluation of atmospheric fields from the ECMWF seasonal forecasts over a 15‐year period. Journal of Climate 18: 16: 3250–3269, 10.1175/JCLI3421.1.

[qj3394-bib-0041] Ollinaho P , Lock SJ , Leutbecher M , Bechtold P , Beljaars ACM , Bozzo A , Forbes RM , Haiden T , Hogan RJ , Sandu I . 2017 Towards process‐level representation of model uncertainties: stochastically perturbed parametrizations in the ECMWF ensemble. Quarterly Journal of the Royal Meteorological Society 143: 702: 408–422, 10.1002/qj.2931.

[qj3394-bib-0042] O'Reilly CH , Woollings T , Zanna L . 2017 The dynamical influence of the Atlantic Multidecadal Oscillation on continental climate. Journal of Climate 30: 18: 7213–7230, 10.1175/JCLI-D-16-0345.1.

[qj3394-bib-0043] Palmer TN , Anderson DLT . 1994 The prospects for seasonal forecasting – a review paper. Quarterly Journal of the Royal Meteorological Society 120: 755–793, 10.1002/qj.49712051802.

[qj3394-bib-0044] Palmer TN , Shutts GJ , Hagedorn R , Doblas‐Reyes F , Jung T , Leutbecher M . 2005 Representing model uncertainty in weather and climate prediction. Annual Review of Earth and Planetary Sciences 33: 1: 163–193, 10.1146/annurev.earth.33.092203.122552.

[qj3394-bib-0045] Saha S , Moorthi S , Wu X , Wang J , Nadiga S , Tripp P , Behringer D , Hou Y‐T , Chuang H‐Y , Iredell M , Ek M , Meng J , Yang R , Mendez MP , van den Dool H , Zhang Q , Wang W , Chen M , Becker E . 2014 The NCEP climate forecast system version 2. Journal of Climate 27: 6: 2185–2208, 10.1175/JCLI-D-12-00823.1.

[qj3394-bib-0046] Scaife AA , Arribas A , Blockley E , Brookshaw A , Clark RT , Dunstone N , Eade R , Fereday D , Folland CK , Gordon M , Hermanson L , Knight JR , Lea DJ , MacLachlan C , Maidens A , Martin M , Peterson AK , Smith D , Vellinga M , Wallace E , Waters J , Williams A . 2014 Skilful long‐range prediction of European and North American winters. Geophysical Research Letters 41: 7: 2514–2519, 10.1002/2014GL059637.

[qj3394-bib-0047] Sheen KL , Smith DM , Dunstone NJ , Eade R , Rowell DP , Vellinga M. . 2017 Skilful prediction of Sahel summer rainfall on inter‐annual and multi‐year timescales. Nature Communications 8: 14966, 10.1038/ncomms14966. PMC5529672 28541288

[qj3394-bib-0048] Shutts GJ . 2005 A kinetic energy backscatter algorithm for use in ensemble prediction systems. Quarterly Journal of the Royal Meteorological Society 131: 3079–3102, 10.1256/qj.04.106.

[qj3394-bib-0049] Vidard A , Anderson DLT , Balmaseda M. . 2007 Impact of ocean observation systems on ocean analysis and seasonal forecasts. Monthly Weather Review 135: 2: 409–429, 10.1175/MWR3310.1.

[qj3394-bib-0050] Wang W , Chen M , Kumar A . 2010 An assessment of the CFS real‐time seasonal forecasts. Weather and Forecasting 25: 3: 950–969, 10.1175/2010WAF2222345.1.

[qj3394-bib-0051] Waters J , Lea DJ , Martin MJ , Mirouze I , Weaver AT , While J . 2015 Implementing a variational data assimilation system in an operational 1/4 degree global ocean model. Quarterly Journal of the Royal Meteorological Society 141: 333–349, 10.1002/qj.2388.

[qj3394-bib-0052] Weisheimer A , Corti S , Palmer TN , Vitart F . 2014 Addressing model error through atmospheric stochastic physical parametrizations: impact on the coupled ECMWF seasonal forecasting system. Philosophical Transactions of the Royal Society A 372: 20130290, 10.1098/rsta.2013.0290. PMC4024238 24842026

[qj3394-bib-0053] Weisheimer A , Schaller N , O'Reilly C , MacLeod DA , Palmer TN . 2017 Atmospheric seasonal forecasts of the twentieth century: multi‐decadal variability in predictive skill of the winter North Atlantic Oscillation (NAO) and their potential value for extreme event attribution. Quarterly Journal of the Royal Meteorological Society 143: 917–926, 10.1002/qj.2976. PMC6686212 31413423

[qj3394-bib-0054] Williams PD . 2012 Climatic impacts of stochastic fluctuations in air–sea fluxes. Geophysical Research Letters 39: L10, 10.1029/2012GL051813.

[qj3394-bib-0055] Williams PD , Howe NJ , Gregory JM , Smith RS , Joshi MM . 2016 Improved climate simulations through a stochastic parameterization of ocean eddies. Journal of Climate 29: 24: 8763–8781, 10.1175/JCLI-D-15-0746.1.

[qj3394-bib-0056] Zadra A , Williams K , Frassoni A , Rixen M , Adames ÁF , Berner J , Bouyssel F , Casati B , Christensen H , Ek MB , Flato G , Huang Y , Judt F , Lin H , Maloney E , Merryfield W , van Niekerk A , Rackow T , Saito K , Wedi N , Yadav P. . 2018 Systematic errors in weather and climate models: nature, origins, and way forward. Bulletin of the American Meteorological Society 99: 4: ES67–ES70, 10.1175/BAMS-D-17-0287.1.

[qj3394-bib-0057] Zanna L . 2012 Forecast skill and predictability of observed Atlantic sea surface temperatures. Journal of Climate 25: 14: 5047–5056, 10.1175/JCLI-D-11-00539.1.

[qj3394-bib-0058] Zanna L , Porta Mana PGL , Anstey J , David T , Bolton T . 2017 Scale‐aware deterministic and stochastic parametrizations of eddy–mean flow interaction. Ocean Modelling 111: 66–80, 10.1016/j.ocemod.2017.01.004.

[qj3394-bib-0059] Zuo H , Balmaseda MA , Mogensen K . 2017 The new eddy‐permitting ORAP5 ocean reanalysis: description, evaluation and uncertainties in climate signals. Climate Dynamics 49: 3: 791–811, 10.1007/s00382-015-2675-1.

