# Supplementary material for: Seasonal to annual ocean forecasting skill and the role of model and observational uncertainty
Source: Q J R Meteorol Soc. 2018 Sep 28;144(715):1947–64. doi: 10.1002/qj.3394 (PMC6472683; doi:10.1002/qj.3394)
Supplement: Supplementary file 1 — Figure S1. Root mean square error of sea surface temperature (K) between the ORAS4 1° reanalysis and its climatological forecast (i.e. mean squared ORAS4 anomalies) for years 1981‐2010 and months a) May, b) June, c) July, d) September, e) December and f) February. Figure S2. Root mean square error of sea surface temperature (K) between the ORAS4 1° reanalysis and its presistence forecast (i.e. using the respective April anomaly for all ten forecast months) for years 1981‐2010 and months a) May, b) June, c) July, d) September, e) December and f) February. Figure S3. Difference in root mean square error of sea surface temperature (K) between REF (Figure 1) and the climatological forecast (Figure S1) for the ORAS4 1° reanalysis for years 1981‐2010 and months a) May, b) June, c) July, d) September, e) December and f) February. Blue shading means REF has a lower RMSE, while red means the climatology of ORAS4 provides a better forecast. Climatology is more accurate than REF mostly in the North Atlantic and along the Kuroshio (from June onwards) due to large model biases in these regions. Figure S4. Same as Figure S3 but for the difference between REF and the per‐sistence forecast (Figure S2). Blue shading means REF has a lower RMSE, while red means that persistence provides a better forecast. While persistence is a better forecast in large areas of the mid latitudes during the first two months, climatology is more accurate thereafter, as it compares in most areas more favorable to REF for July{February. Persistence is more accurate than REF mostly in the North Atlantic and also the Kuroshio due to large model biases in these regions. Figure S5. Same as Figure S3 but for upper 300m ocean heat content (J/m 2). Compared to sea surface temperature in Figure S3 REF remains the more skilful forecast for much longer. Climatology is more accurate than REF mostly in the North Atlantic and along the Kuroshio (from September onwards) due to large model biases in these regions. Figur [file QJ-144-1947-s001.pdf]

Supporting Information
Seasonal to annual ocean forecasting skill and the role of model and observational uncertainty

Stephan Juricke, Dave MacLeod, Antje Weisheimer,
Laure Zanna, and Tim Palmer

July 5, 2018

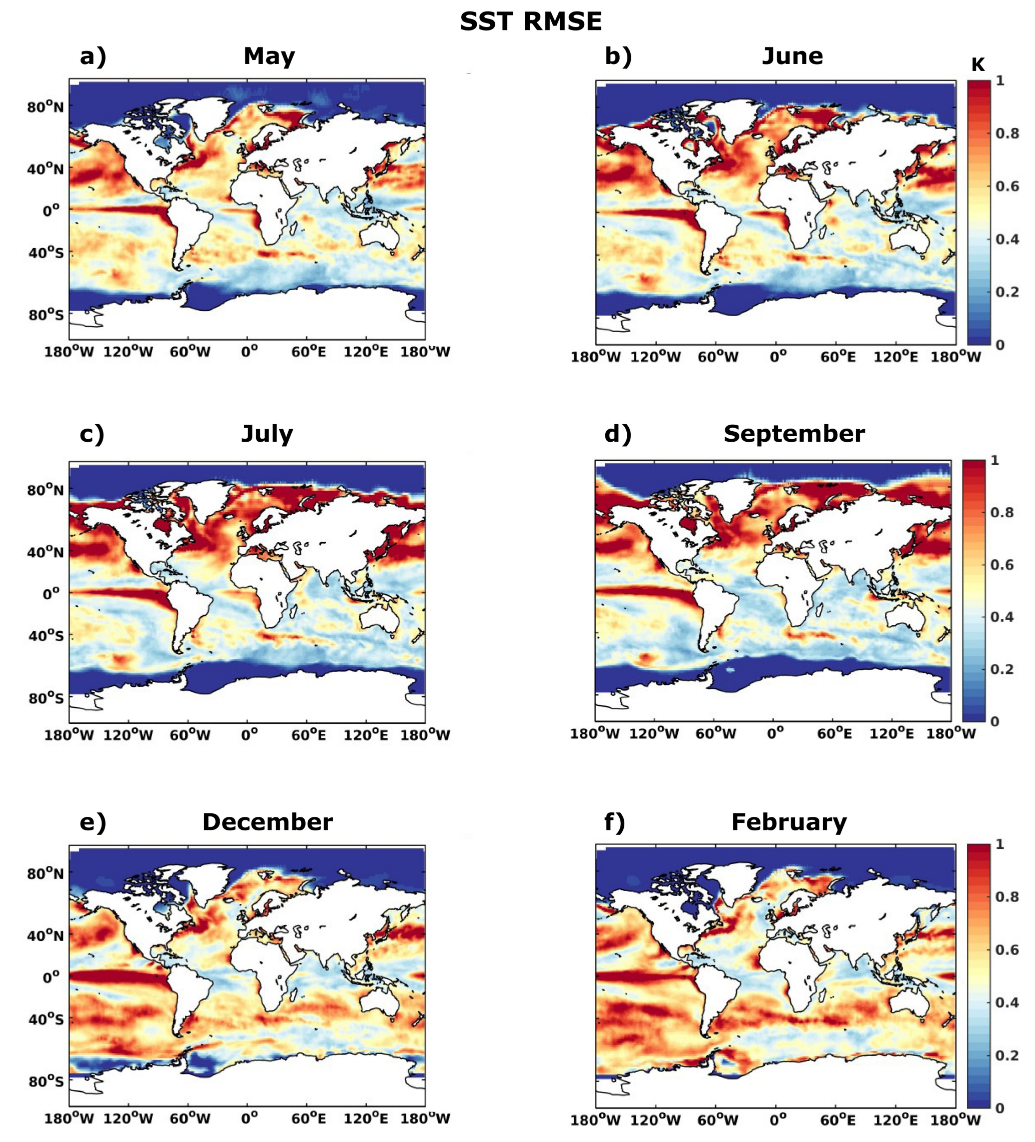

Figure S1: Root mean square error of sea surface temperature (K) between the ORAS4 1° reanalysis and its climatological forecast (i.e. mean squared ORAS4 anomalies) for years 1981-2010 and months a) May, b) June, c) July, d) September, e) December and f) February.

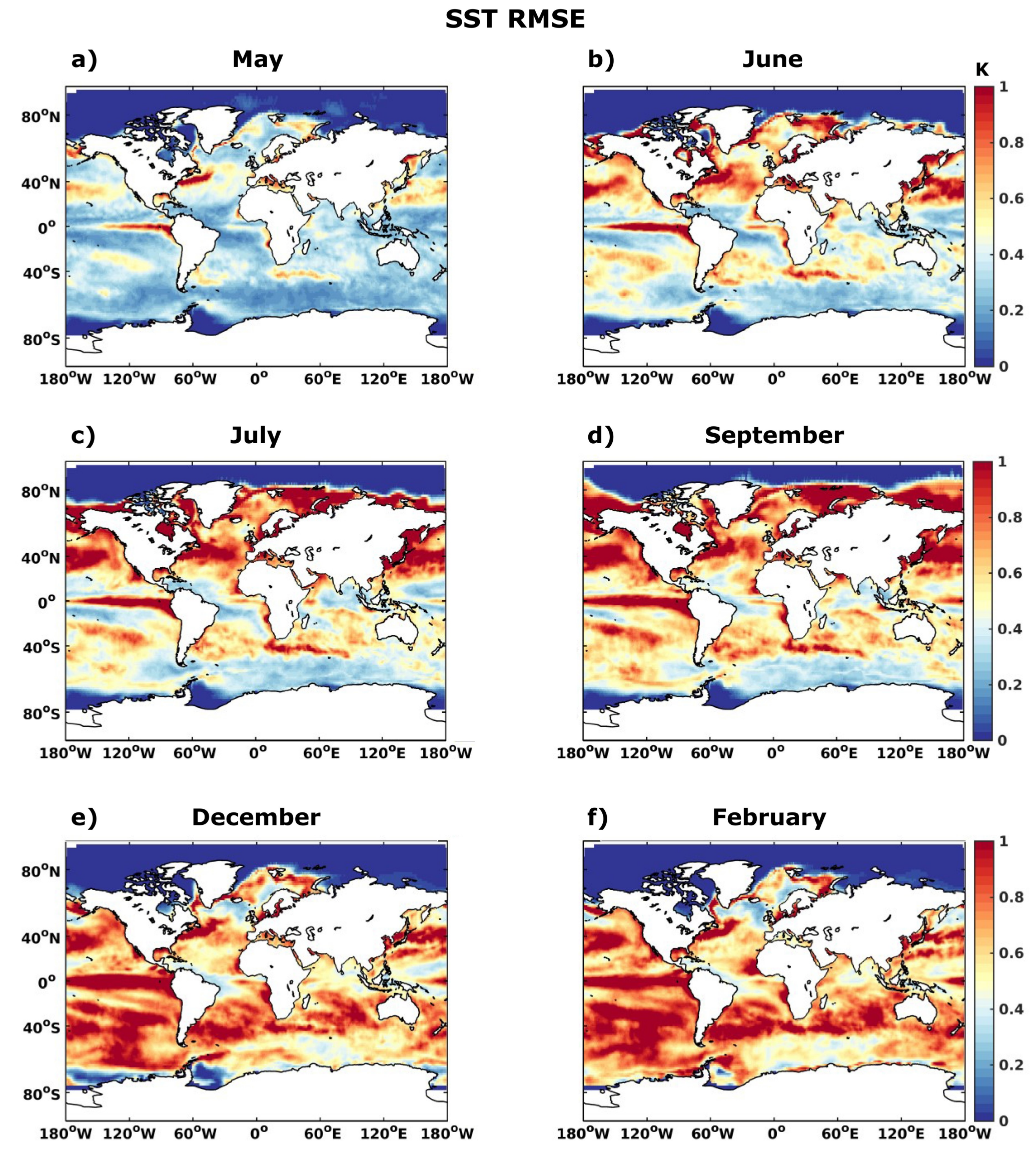

Figure S2: Root mean square error of sea surface temperature (K) between the ORAS4 1° reanalysis and its presistence forecast (i.e. using the respective April anomaly for all ten forecast months) for years 1981-2010 and months a) May, b) June, c) July, d) September, e) December and f) February.

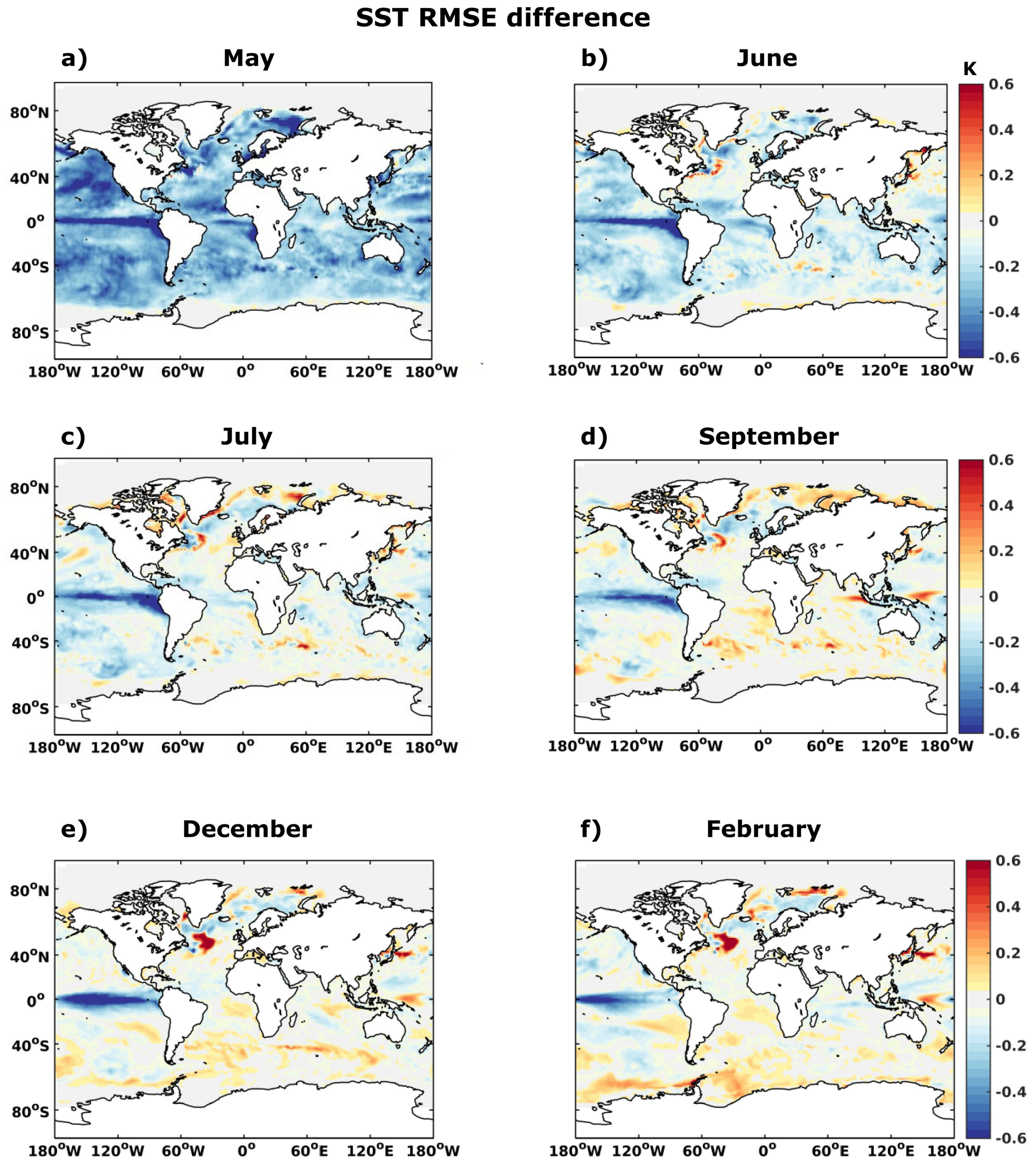

Figure S3: Difference in root mean square error of sea surface temperature (K) between REF (Figure 2) and the climatological forecast (Figure S1) for the ORAS4 1° reanalysis for years 1981-2010 and months a) May, b) June, c) July, d) September, e) December and f) February. Blue shading means REF has a lower RMSE, while red means the climatology of ORAS4 provides a better forecast. Climatology is more accurate than REF mostly in the North Atlantic and along the Kuroshio (from June onwards) due to large model biases in these regions.

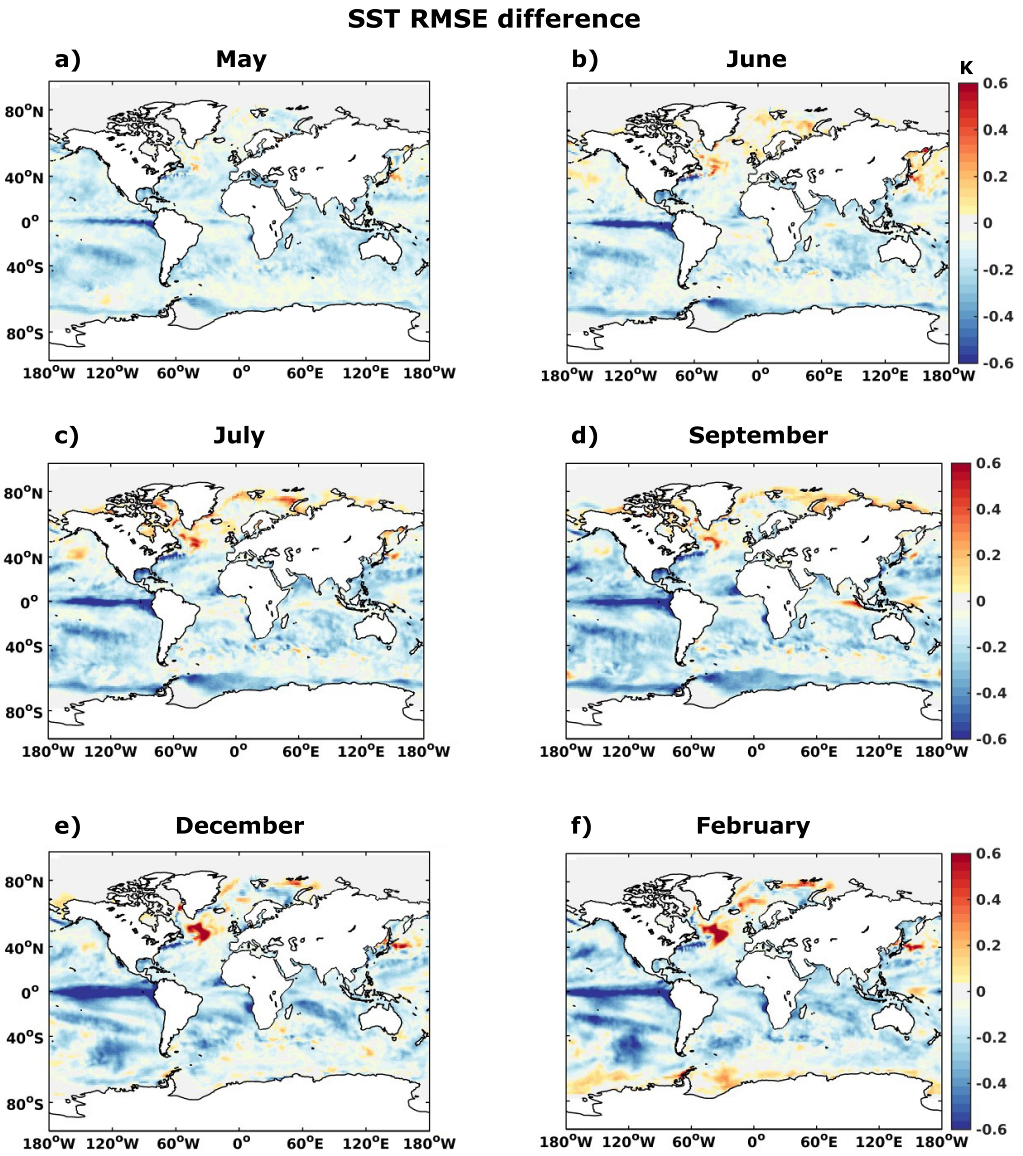

Figure S4: Same as Figure S3 but for the difference between REF and the persistence forecast (Figure S2). Blue shading means REF has a lower RMSE, while red means that persistence provides a better forecast. While persistence is a better forecast in large areas of the mid latitudes during the first two months, climatology is more accurate thereafter, as it compares in most areas more favorable to REF for July–February. Persistence is more accurate than REF mostly in the North Atlantic and also the Kuroshio due to large model biases in these regions.

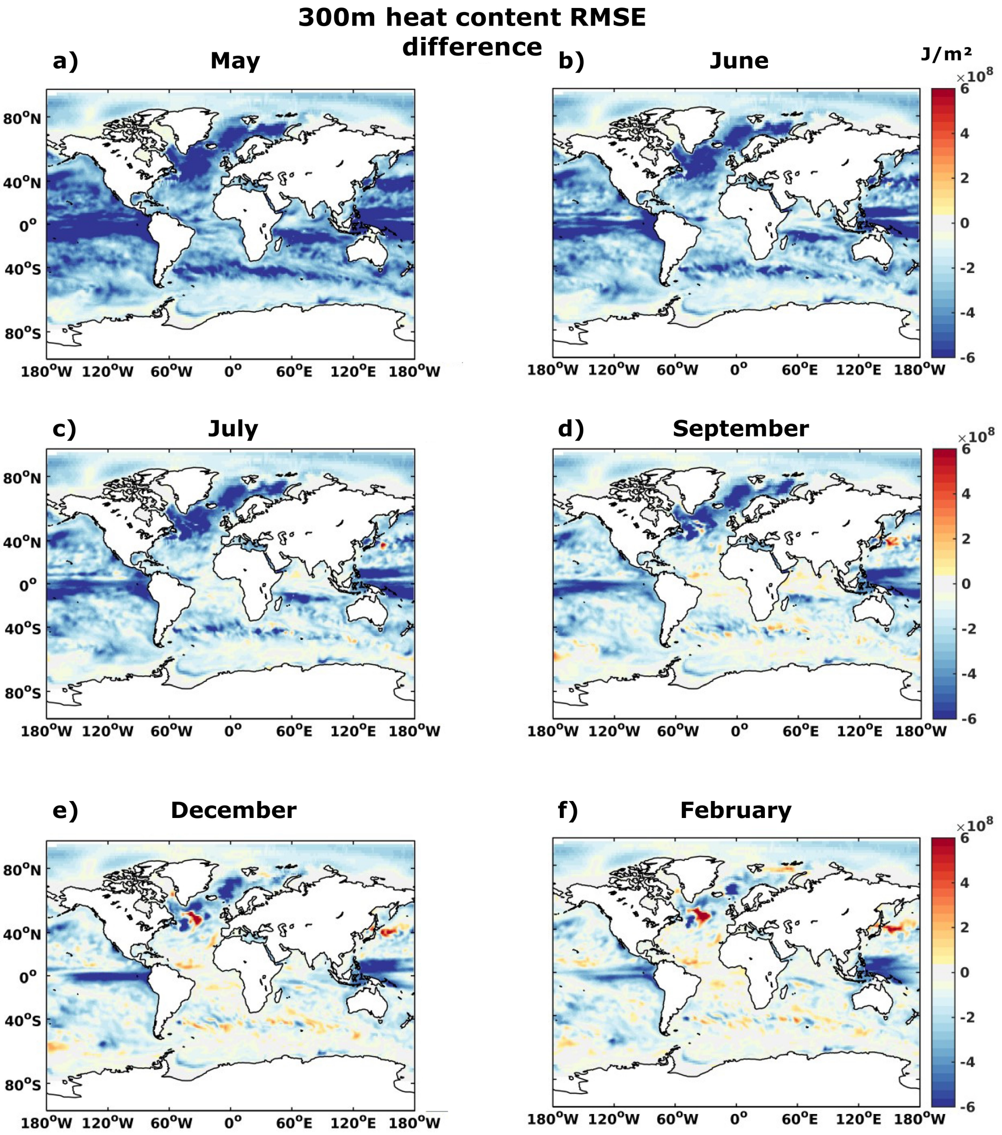

Figure S5: Same as Figure S3 but for upper 300m ocean heat content (J/m^2). Compared to sea surface temperature in Figure S3 REF remains the more skilful forecast for much longer. Climatology is more accurate than REF mostly in the North Atlantic and along the Kuroshio (from September onwards) due to large model biases in these regions.

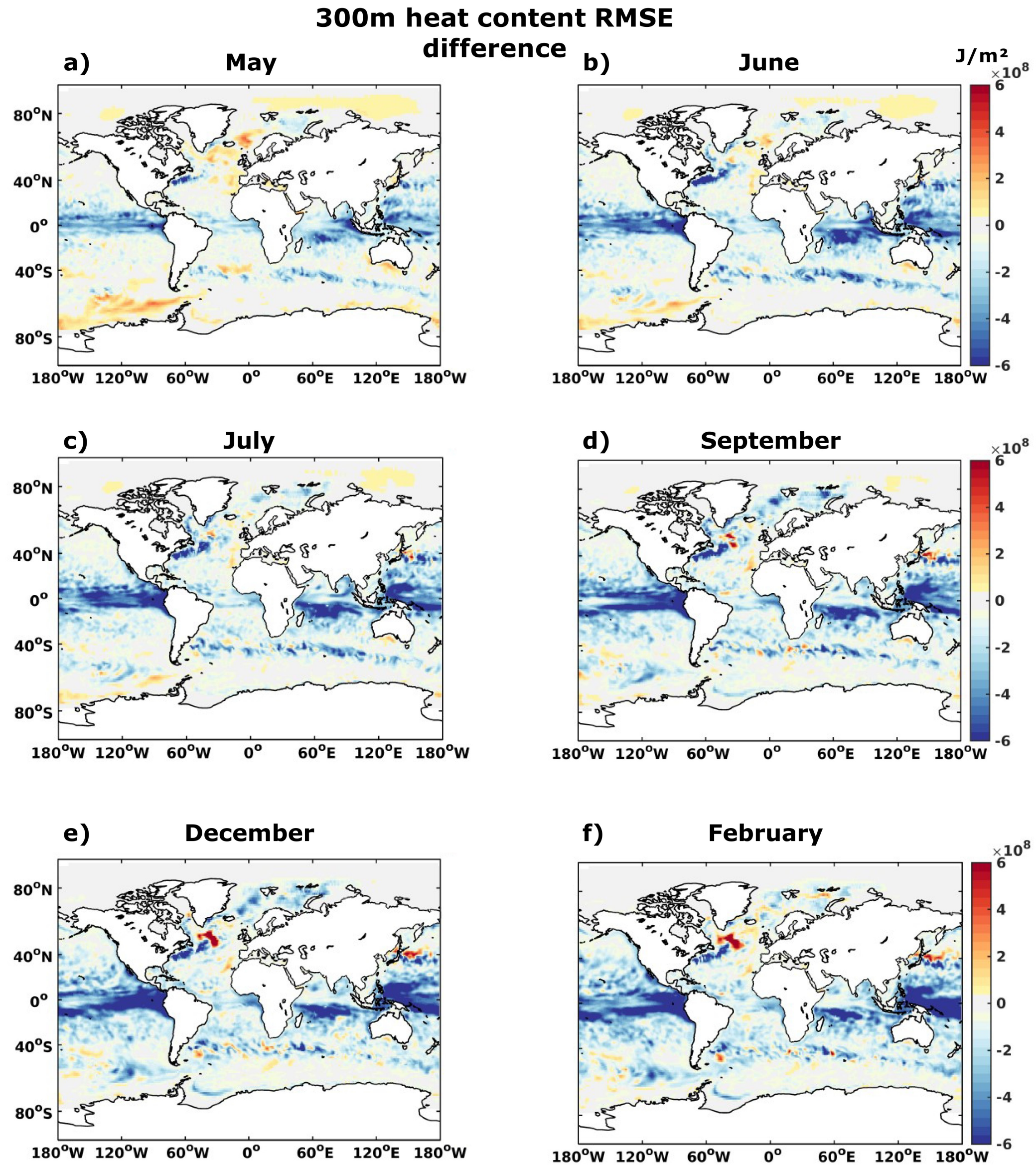

Figure S6: Same as Figure S4 but for upper 300m ocean heat content (J/m^2). Although persistence of heat content lasts a little longer than for sea surface temperature (compare RMSE difference progression through the year with Figure S4) the forecast is nearly everywhere less skilful than REF already after one month, and less skilful than the climatology for large areas from December onwards. Persistence is more accurate than REF mostly in the North Atlantic and also the Kuroshio (from July onwards) due to large model biases in these regions.

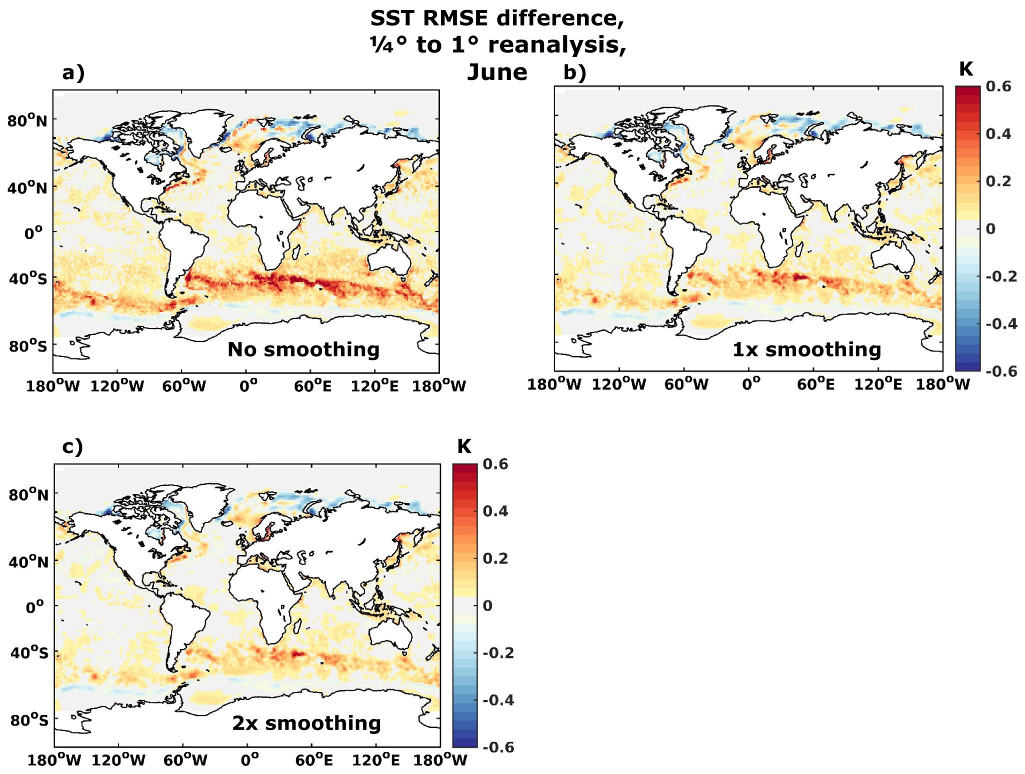

Figure S7: Effects of spatial smoothing on sea surface temperature root mean square error difference between REF verified against ORAP5 (1/4)° reanalysis and REF verified against OARS4 1° reanalysis ($\mathrm{RMSE}_{\mathrm{ORAP5}} - \mathrm{RMSE}_{\mathrm{ORAS4}}$) for June of years 1981-2010: a) no smoothing; b) smoothing filter applied once; c) smoothing filter applied twice. The smoothing filter uses a nine-point stencil to compute a weighted mean. The central grid point has weight 1, the four direct neighbours have weight 0.5 and the diagonal neighbours have weight 0.3. Smoothing is performed on the REF grid for the SST fields from REF, ORAS4, and ORAP5. In the mid latitudes the smoothing operates approximately over the surrounding 100km, applying the smoothing twice increases this to around 200km. In the tropics this distance is smaller because of the telescoping of the grid. It is also smaller in the polar regions. Generally, the RMSE estimate of REF is larger when using ORAP5 as reference, signified by the red shading. As the smoothing is increased the difference between the error estimates for the two reanalyses is reduced but remains substantial in the mid latitudes. This implies that the difference does only to some degree originate from the interpolation of an eddy permitting reanalysis to a grid that does not resolve eddies.

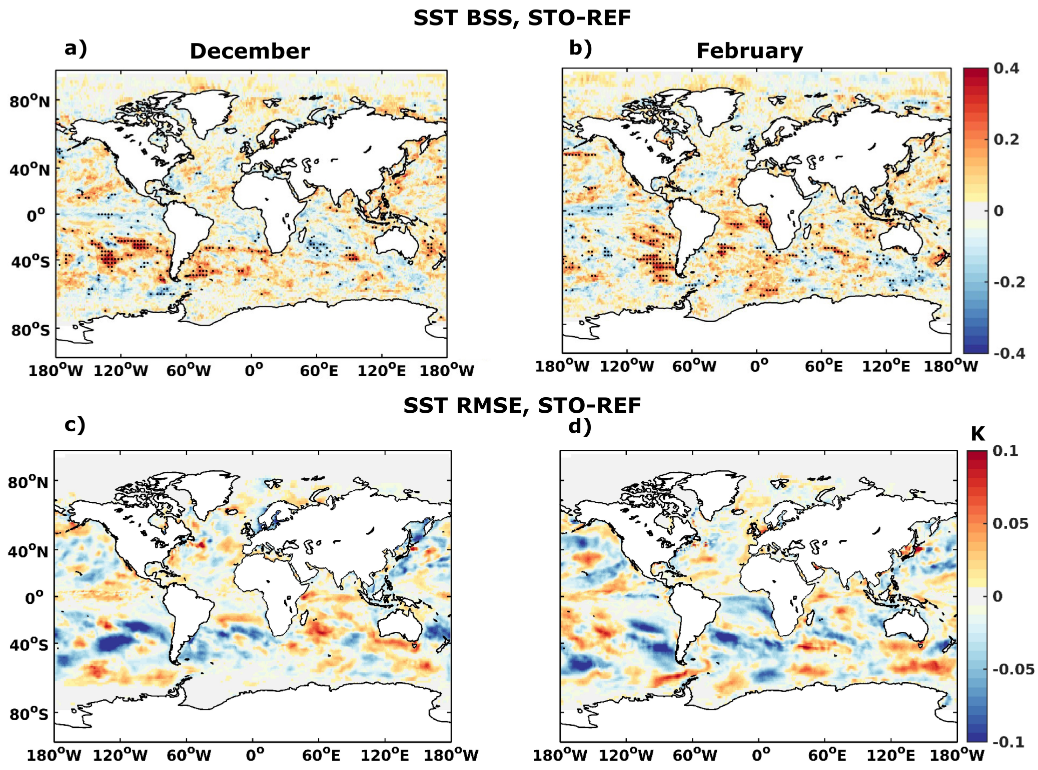

Figure S8: Difference in sea surface temperature (top) Brier skill score as well as (bottom) root mean square error between STO and REF for start dates 1981-2010 and months a) and c) December and b) and d) February, referenced to ORAS4 1° reanalysis. Stippled areas for BSS indicate significant differences according to where the value of the upper quartile of the respective lower score (e.g. of REF) does not reach the value of the lower quartile of the respective higher score (e.g. of STO), with quartiles generated by the 1000 sample bootstrapping distributions (with replacement) for STO and REF. Similar significance estimates are found when considering only those differences where STO lies outside the 90% confidence interval of REF. Areas of significant BSS increase (decrease) in STO, i.e. stippled red shading (blue shading) in a) and b), coincide with those areas where the RMSE for STO is reduced (increased) compared to REF, i.e. blue shading (red shading) in c) and d). This indicates that significant changes in BSS are to some extent caused by the changes in RMSE.
